# Comparative Evaluation of the Antibacterial and Antitumor Activities of Marine Alkaloid 3,10-Dibromofascaplysin

**DOI:** 10.3390/md23020068

**Published:** 2025-02-06

**Authors:** Maxim E. Zhidkov, Polina A. Smirnova, Natalia E. Grammatikova, Elena B. Isakova, Andrey E. Shchekotikhin, Olga N. Styshova, Anna A. Klimovich, Aleksandr M. Popov

**Affiliations:** 1 Department of Chemistry and Materials, Institute of High Technologies and Advanced Materials, FEFU Campus, Far Eastern Federal University, Ajax Bay 10, Russky Island, Vladivostok 690922, Russia; smirnova_pa@dvfu.ru; 2 Laboratory of Chemical Transformation of Antibiotics, Gause Institute of New Antibiotics, Moscow 119021, Russia; ngrammatikova@yandex.ru (N.E.G.); ebisakova@yandex.ru (E.B.I.); shchekotikhin@mail.ru (A.E.S.); 3 Departments of Biotechnology and Marine Natural Compounds Chemistry, G.B. Elyakov Pacific Institute of Bioorganic Chemistry, Far Eastern Branch of The Russian Academy of Sciences, Vladivostok 690922, Russia; krivoshapkoon@mail.ru (O.N.S.); annaklim_1991@mail.ru (A.A.K.); popovam@piboc.dvo.ru (A.M.P.)

**Keywords:** fascaplysin derivatives, antibacterial activity, antitumor efficacy, 3,10-dibromofascaplysin

## Abstract

Fascaplysins form a group of marine natural products with unique cationic five-ring coplanar backbone. Native fascaplysin exhibits a broad spectrum of bioactivities, among which the cytotoxic activity has been the most investigated. Several fascaplysin derivatives have more selective biological effects and are promising as lead compounds. Thus, the introduction of a substituent at C-9 of fascaplysin leads to a strong increase in its antimicrobial properties. Here, a comparative assessment of the antimicrobial activity of synthetic analogs of the marine alkaloids 3-bromofascaplysin, 10-bromofascaplysin, and 3,10-dibromofascaplysin, along with some of their isomers and analogs, was carried out against a panel of Gram-positive bacteria in vitro. For the first time, a significant increase in the antimicrobial activity of fascaplysin was observed when a substituent was introduced at C-3. The introduction of two bromine atoms at C-2 and C-9 enhances the antimicrobial properties by 4 to 16 times, depending on the tested strain. Evaluation of the antimicrobial potential in vivo showed that fascaplysin and 3,10-dibromofascaplysin had comparable efficacy in the mouse staphylococcal sepsis model. Additionally, 3,10-dibromofascaplysin demonstrated a strong and reliable antitumor effect in vivo on the Ehrlich carcinoma inoculated subcutaneously, with a value of tumor growth inhibition by 49.2% 20 days after treatment. However, further studies on alternative chemical modifications of fascaplysin are needed to improve its chemotherapeutic properties.

## 1. Introduction

**Figure 1:**
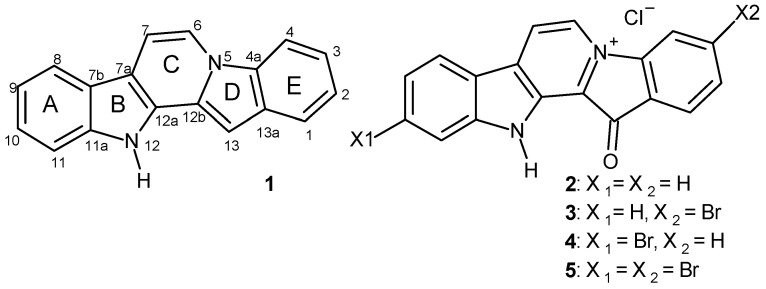
Structures of 12*H*-pyrido[1,2*-a*:3,4-*b′*]diindole (**1**) and fascaplysins **2**–**5**.

The 12*H*-pyrido [1,2-*a*:3,4-*b*′]diindole ring system (**1**) forms the framework of several marine alkaloids, including fascaplysin (**2**), 3-bromofascaplysin (**3**), 10-bromofascaplysin (**4**), and 3,10-dibromofascaplysin (**5**) (Figure 1). The red pigment fascaplysin, which was isolated first in 1988 from the sponge *Fascaplysinopsis* sp., is the most investigated [1]. Upon its isolation, a broad spectrum of biological activities were demonstrated for this compound, including the cytotoxic effects on tumor cells, antibacterial activity against both Gram-positive and Gram-negative bacteria, and antifungal properties. In recent decades, significant progress has been made in the development of methods for the synthesis of fascaplysin and its derivatives, as well as in understanding the mechanisms underlying its cytotoxic activity [2]. In the early 2000s, the ability of fascaplysin to intercalate into DNA and selectively inhibit the cyclin-dependent kinase 4 was demonstrated [3,4]. This, in turn, led to the development of non-planar analogs of fascaplysin [5,6,7,8,9,10]. Later, it was found that fascaplysin induces a complex cascade of interactions that results in apoptotic cell death [11]. The initial event involves mitochondrial damage, leading to the disruption of mitochondrial membrane potential (Δψm). This leads to the opening of mitochondrial permeability transition pores (PTPs), which release pro-apoptotic proteins from the mitochondria to cytosol, a decrease in ATP levels, an increase in the reactive oxygen species (ROS) level within the cells, and the triggering of autophagy [12]. ROS, in turn, cause DNA damage and activate repair mechanisms. Fascaplysin also inhibits the PI3K/AKT/mTOR signaling pathway, at least partly, through the inhibition of VEGFR3, VEGFR2, and TRKA [13,14]. Disruption of this critical signaling cascade contributes to the antiangiogenic properties of fascaplysin and partially underlies its apoptotic effects via the inhibition of survivin synthesis. However, numerous experiments on various in vivo models have shown the futility of utilizing fascaplysin as an anticancer drug [13,14,15,16,17]. Despite this, efforts to develop antitumor drugs based on the fascaplysin scaffold are ongoing [18,19].

**Figure 2:**
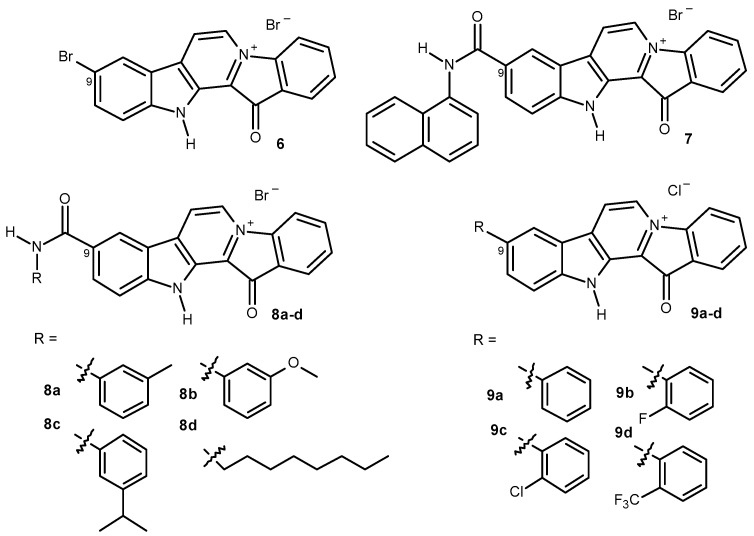
The structures of fascaplysin derivatives **6**–**9** with high antibacterial activity.

Another direction for fascaplysin optimization is the development of novel antibacterial candidates. The spread of multi-drug-resistant (MDR) pathogenic bacteria presents a significant challenge to global health systems [20]. Therefore, there is an urgent need to explore new molecular scaffolds that exhibit potent antibacterial properties or act on novel targets in bacterial cells [21]. It has been shown that derivatives **6** and **7** exhibit excellent antimicrobial activity against methicillin-resistant *Staphylococcus aureus* (MRSA) and effectively suppress the formation of its biofilms (Figure 2) [22]. Compound **7** displays low hemolytic activity and cytotoxicity to mammalian cells, effectively disrupts bacterial cell walls, and damages bacterial cell membranes in vitro. In a mouse peritonitis model in vivo, it also gave a 20% survival rate at a dose of 10 mg/kg. Subsequent optimization was focused on the modification of the amine residue of compound **7** [23]. As a result, a series of improved derivatives **8a**–**c** were synthesized. These compounds, in addition to exhibiting high antibacterial activity against Gram-positive bacteria, showed no significant tendency to induce bacterial resistance. Furthermore, compound **8** promoted the polymerization of the filamentous temperature-sensitive mutant Z (FtsZ) at 4 µg/mL and decreased the GTPase activity of FtsZ (for compound **8c**, there was a 40% reduction at the concentration of 0.32 μg/mL), which is at least partially related with the antibacterial activity of these compounds. FtsZ, an initiator of bacterial cell division, has become one of the most attractive protein targets for the development of novel antibacterial agents. This is due to its high conservation and the low sequence similarity to homologous tubulins of eukaryotes [24,25,26,27,28]. It has also been reported that fascaplysin derivatives can promote the oligomerization of β-amyloid, a protein involved in the pathogenesis of Alzheimer’s disease, suggesting that fascaplysin derivatives may act as promotors of protein aggregation [29,30].

Another series of fascaplysin derivatives **9a**–**c** has been designed and synthesized via Suzuki–Miyaura cross-coupling [31]. Some compounds exhibited potent bactericidal activities against a panel of Gram-positive (MIC = 0.024–6.25 μg/mL) and Gram-negative (MIC = 1.56–12.5 μg/mL) bacteria. However, additional studies of the interaction of the obtained compounds with FtsZ via molecular docking and molecular dynamics methods yielded contradictory and ambiguous results. Unfortunately, these compounds proved to be more cytotoxic compared to the derivatives of series **7**, with their IC_50_ values being comparable to their MIC values. In previous studies, we assessed the therapeutic potential of compound **9a**, as well as a series of its analogs [19]. 9-Phenylfascaplysin (**9a**) was found to be more effective than the reference antibiotic vancomycin in vivo. However, its ED_50_ value was similar to that of unsubstituted fascaplysin (**2**), despite being significantly more active than the paternal alkaloid in vitro. When tested for antitumor activity against Ehrlich carcinoma in mice, compound **9a** showed substantial effectiveness during the initial stages of treatment, but the antitumor effect decreased rapidly after its course. Additionally, the low water solubility of this compound posed a significant challenge in vivo. The introduction of polar fragments to the phenyl substituent capable of forming hydrogen bonds resulted in a sharp decrease in both antibacterial and antitumor activity in vitro. Increasing the volume of the lipophilic substituent also did not lead to an additional increase in potency in vitro. These data reveal the limited potential of derivatives at C-9, highlighting the need to expand the diversity of fascaplysin derivatives studied for antibacterial activity in order to identify novel lead compounds for future drug development.

Among the known fascaplysin derivatives, its natural bromo derivatives have been studied the most. 3-Bromo and 10-bromofascaplysins (**3**, **4**) exhibit antiproliferative activity at submicromolar concentrations [32]. This effect was mediated through the induction of caspase-8-, -9-, and -3-dependent apoptosis. The antitumor effects of 3-bromofascaplysin and 10-bromofascaplysin were evaluated on glioma C6 cells in vitro. The antiproliferative activity of 3-bromofascaplysin was found to be higher than of fascaplysin and 10-bromofascaplysin [33]. Remarkably, 3,10-dibromofascaplysin (**5**) was able to suppress the cell metabolism at non-cytotoxic concentrations [34]. Further studies aimed at determining the mechanism of its antiproliferative effect in myeloid leukemia cells revealed that compound **5** activates the transcription factor E2F1 and decreases the expression of several genes responsible for cancer cell survival [35]. Additionally, in human prostate cancer cells, JNK1/2 was identified as one of the primary molecular targets of **5**. Furthermore, 3,10-dibromofascaplysin was shown to synergize with the PARP-inhibitor olaparib, presumably due to the induction of ROS production and consequent oxidative DNA damage mediated by the drug [36]. Moreover, compound **5** enhanced the potency of well-established anticancer drugs such as cytarabine, cisplatin, and carboplatin, as well as docetaxel and cabazitaxel [34,35].

In this paper, we present the results of the evaluation of the antibacterial activity of synthetic bromo derivatives of fascaplysin **3**–**5** and several analogs in vitro. Based on these data and previously obtained results, the antibacterial and antitumor effects of 3,10-dibromofascaplysin (**5**) were evaluated in vivo.

## 2. Results and Discussion

### 2.1. Preparation of Fascaplysins **3**–**5**, Its Isomers, and Some Analogs

To date, a lot of methods for the synthesis of fascaplysin and its derivatives and analogs have been reported [37,38,39,40,41,42,43,44,45,46,47]. The two-step procedure suggested by Zhu et al. is the most suitable for the preparation of fascaplysin derivatives from substituted tryptamines and acetophenones [45]. This approach is based on the cascade coupling protocol, which includes the sequential iodination of ortho-halogen-substituted acetophenone, the Kornblum oxidation of the intermediate in the presence of DMSO to phenylglyoxal, followed by its Pictet–Spengler condensation with corresponding tryptamine, and finally the oxidation of the resulting semi-product. This method was adapted to prepare fascaplysin and all its derivatives used in this study, with the exception of 10-bromofascaplysin (**4**), which was synthesized as described in [48].

**Scheme 1:**
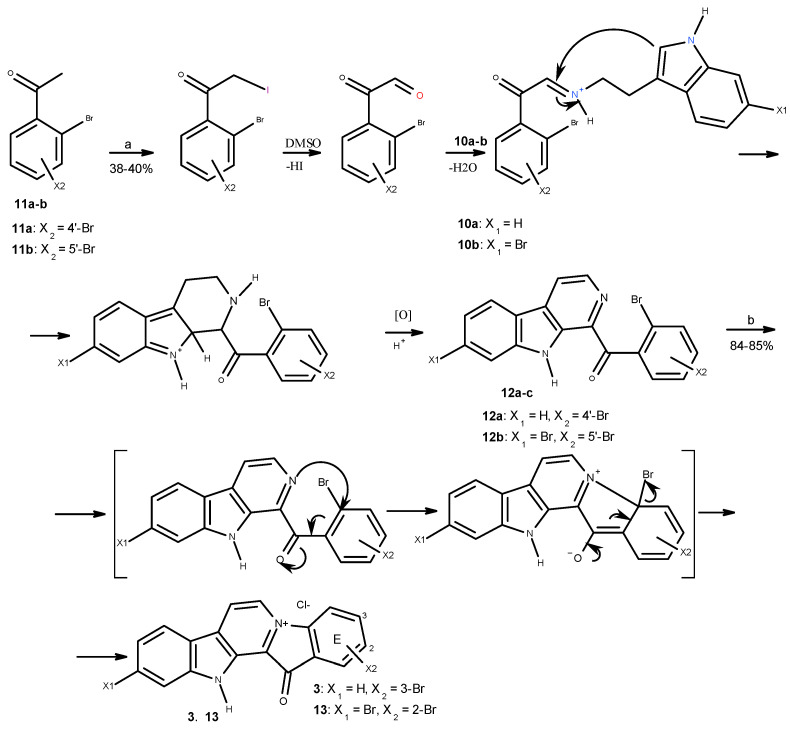
Preparation of 3-bromofasaplysin (**3**) and 2,10-dibromofascaplysin (**13**). Reagents and conditions: (a) I_2_ (0.8 equiv.), DMSO, 110 °C, 4 h; (b) 220 °C, 40 min, and HCl (aq).

To prepare 3-bromofascaplysin (**3**), the original method was applied using tryptamine (**10a**) and 2,4-dibromoacetophenone (**11a**). To synthesize 2,10-dibromofascaplysin (**13**), starting compound **10a** was replaced by 6-bromotryptamine (**10b**), which was obtained using the previously described method [49]. The condensation of 6-bromotryptamine (**10b**) with 2,5-dibromoacetophenone (**11b**) in the cascade coupling protocol yielded compound **12b** in moderate yield (Scheme 1). Compounds **12a** and **12b** were then subjected to high-temperature quaternization, which occurs via an intramolecular variant of the mechanism of nucleophilic aromatic substitution. This is confirmed by the fact that the presence of the electron-withdrawing carbonyl group of the benzoyl fragment is critical for this reaction to proceed. When it is replaced by the -CH_2_- fragment, quaternization does not occur under the given conditions. Next, in the resulting salts, the bromide anion was replaced by a chloride anion with the action of hydrochloric acid [40].

**Scheme 2:**
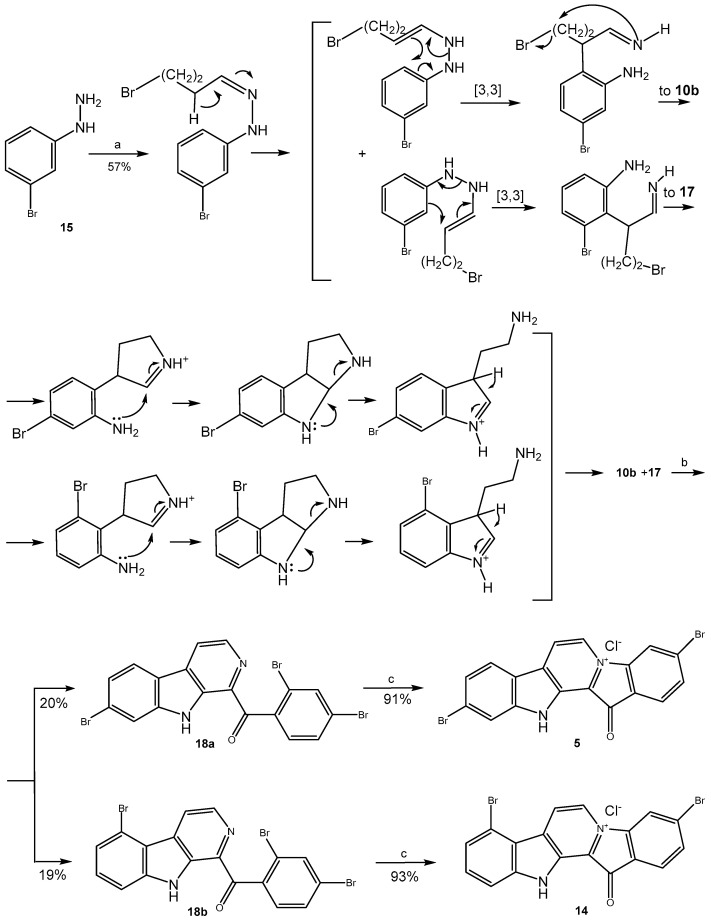
Synthesis of 3,10-dibromofascaplysin (**5**) and its isomer **14**. Reagents and conditions: (a) 4-bromobutanal (**16**, 4.0 equiv.), EtOH, H_2_O, autoclave, 150 °C, 1 h; (b) 2,4-dibromoacetophenone (**11a**) (1 equiv.), I_2_ (0.8 equiv.), DMSO, 110 °C, 1 h, then tryptamines **10b**, **17** (1.0 equiv.), DMSO, 110 °C, 4 h; (c) 220 °C, 15 min, then HCl (aq).

To apply this synthetic approach for the synthesis of 3,10-dibromofascaplysin (**5**) and 3,8-dibromofascaplysin (**14**), the reaction between 3-bromophenylhydrazine (**15**) and 4-bromobutanal (**16**) in an autoclave at 150 °C was used to prepare the mixture of 6-bromotryptamine (**10b**) and 4-bromotryptamine (**17**) (Scheme 2). This transformation is based on a known method for obtaining tryptamines from phenylhydrazines and 4-halobutanals and includes the Fischer indole synthesis as a key step in the reaction. Therefore, the usage of meta-substituted phenylhydrazine as the starting compound ensures annulation of the pyrrole ring on both sides of the hydrazo group and the formation of a mixture of two isomeric tryptamines **10b** and **17** in approximately equal proportions. The obtained mixture and 2,4-dibromoacetophenone (**11a**) were coupled according to the protocol described above. After chromatography separation and purification, 1-benzoyl-β-carbolines **18a** and **18b** were subsequently transformed to 3,10-dibromofascaplysin (**5**) and its isomer **14** in accordance with the mechanism shown in Scheme 1 [34].

**Scheme 3:**
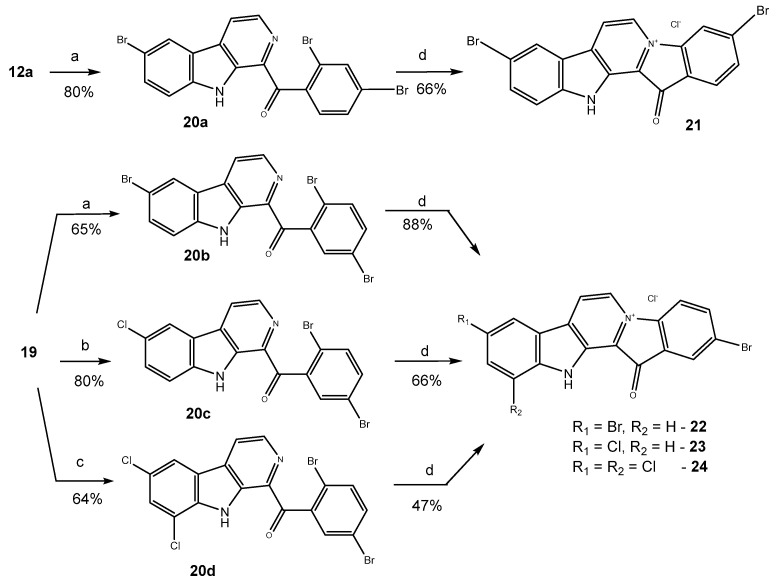
Synthesis of a series of disubstituted fascaplysin derivatives **21**–**24**. Reagents and conditions: (a) NBS (2.0 equiv.), AcOH, 90 °C, 1 h; (b) Cl_2_ (excess), AcOH, r.t., 2 h; (c) Cl_2_ (excess), AcOH, 60 °C, 1 h; (d) 200 °C, 1–2 h, then HCl (aq).

Previously, we demonstrated that the direct bromination, chlorination, and iodination of 1-(2′-bromobenzoyl)-β-carboline resulted in the corresponding halogenated derivatives with yields ranging from 66% to 70% [50]. It was also shown that carrying out the chlorination and bromination reaction at a higher temperature led to the preparation of dihalogenated derivatives of β-carboline containing substituents at C-6 and C-8. The combination of the method developed by Zhu et al. with the electrophilic substitution of intermediate β-carbolines allowed for the synthesis of a series of disubstituted derivatives of fascaplysin with the substitutes at rings A and E. To evaluate the effect of the substitution of fascaplysin at C-3 and C-9, we performed the bromination of 1-(2′,4′-dibromobenzoyl)-β-carboline (**12a**) and the conversion of corresponding 1-benzoyl-β-carboline to 3,9-dibromofascaplysin (**21**). Additionally, the bromination and chlorination of 1-(2′,5′-dibromobenzoyl)-β-carboline (**19**), followed by the cyclization of the obtained products, gave fascaplysin derivatives **22**–**24** (Scheme 3).

### 2.2. Antibacterial and Antiproliferative Activities of 3-Bromo-, 10-Bromo-, and 3,10-Dibromofascaplysins, Their Isomers, and Analogs In Vitro

The antibacterial activity of synthetic fascaplysin (**2**), 3-bromofascaplysin (**3**), 10-bromofascaplysin (**4**), 3,10-dibromofascaplysin, (**5**) and their derivatives and analogs **13**, **14**, **21**–**24** was evaluated on a broad panel of pathogenic bacteria using the broth microdilution method. Gram-positive bacteria are presented by four *Staphylococcus aureus* strains (including two methicillin-resistant strains—MRSA), *Bacillus cereus*, a *Staphylococcus epidermidis* strain, four *Enterococcus* spp. strains (including two vancomycin-resistant strains—VRE), and *Mycobacterium smegmatis*. Gram-negative bacteria are presented by *Escherichia coli*. The screening panel includes ATCC reference stains of the main types of bacteria and antibiotic-resistant isolates, which refers to the priority pathogens list of the World Health Organization. Vancomycin (Van) and rifampicin (Rif) were used as positive controls for antibacterial screening. The results of the comparative tests of antibacterial potency are shown in Table 1. For comparison, the activity of 9-phenylfascaplysin (**9a**) against the same pathogen panel under similar conditions is also presented from [19].

The data presented in Table 1 show that the introduction of a single bromine atom to the structure of fascaplysin at C-3 (**3**) and C-10 (**4**) leads to an increase in antimicrobial potency against Gram-positive bacteria, with the modification at C-3 resulting in a significantly stronger effect. The antimicrobial activity profile of compound **3** is surprisingly similar to that of previously identified compound **9a**: compared to fascaplysin (**2**), compound **3** shows increased activity against *E. faecalis*, but with a sharp decrease in activity against *E. faecium* and *M. smegmatis*. No change in antimicrobial activity compared to fascaplysin (**2**) was observed for *E. coli*.

Surprisingly, the introduction of two bromine atoms to the fascaplysin structure at C-3 and C-10 did not affect the antimicrobial potency, and the activity of dibromo derivative **5** was almost identical to that of monobromo analog **3**. Shifting the bromine atom from C-3 to C-2 of ring E (compound **13**) or from C-10 to C-8 or C-9 of ring A (compounds **14** and **21**, respectively) generally resulted in an additional increase in activity against the most sensitive strains and helped circumvent the resistance of *E. faecium*. Finally, the combination of two bromine atoms at C- 2 and C-9 led to the most active isomer **22**, which was 4 to 16 times more active than isomeric 3,10-dibromofascaplysin (**5**). Its chlorine-containing analog **23** showed almost identical potency with minor differences against some of the strains studied. An additional chlorine atom at C-11 (compound **24**) led to a decrease in antimicrobial activity.

However, it should be noted that derivatives **13**, **14**, and **21**–**23** exhibit lower aqueous solubility compared to paternal fascaplysin (**2**). In contrast, 3-bromofascaplysin has assessable solubility but exhibits relatively high cytotoxicity [33]. Given that 3,10-dibromofascaplysin has low cytotoxicity for mammalian cells [50] and relatively higher solubility compared to other disubstituted derivatives of fascaplysin, compound **5** was selected for testing its antibacterial and antitumor properties in vivo.

### 2.3. Study of the Therapeutic Potential of 3,10-Dibromofascaplysin In Vivo

#### 2.3.1. Antibacterial Efficacy of 3,10-Dibromofascaplysin

**Figure 3:**
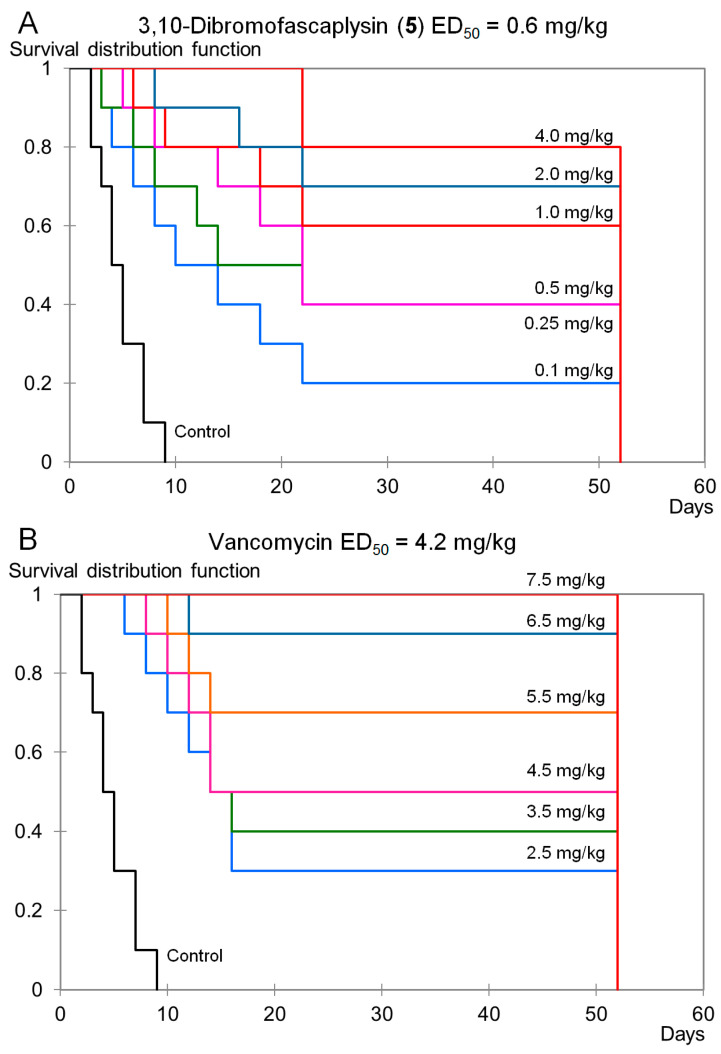
Survival curves (Kaplan–Meier) of mice infected with *S. aureus* after intravenous (i.v.) administration of 3,10-dibromofascaplysin (**A**) and vancomycin (**B**). Mice (*n* = 10 for each group) were treated 1 h after infection via corresponding single doses of the drugs shown on the curves.

For the selected 3,10-dibromofascaplysin (**5**), its antibacterial efficacy was assessed in comparison with the reference antibiotic vancomycin (Van) using a mouse staphylococcal sepsis model with a single-dose intravenous (i.v.) administration. This study’s results are presented in Figure 3. Compound **5** exhibited lower water solubility than fascaplysin (**2**), which posed challenges for its testing in vivo. However, the addition of 10–20% ethanol or PEG600 as solubilizing agents significantly improved the aqueous solubility of compound **5**. During this study, animal mortality was observed in the treatment groups up to day 22 (Figure 3A,B), with the survival observation period extended to 30 days after the last recorded death, at which point all the remaining mice were euthanized.

The results of this study showed that the survival rate of mice infected with *S. aureus* increased after a single-dose i.v. administration of compound **5** or Van in a clear dose-dependent manner (Figure 3). It should be noted that even at the minimal dose (0.1 mg/kg), derivative (**5**) provided a significant antibacterial effect and nearly doubled the median survival rate. However, 100% survival of the experimental animals was not achieved at the highest dose of **5** (4.0 mg/kg). Based on the survival data, the effective dose (ED_50_) values for the tested drugs were calculated (Figure 3). The ED_50_ value of 3,10-dibromofascaplysin (**5**) was almost seven times lower than that of the reference vancomycin. Surprisingly, the ED_50_ value for compound **5** was similar to that of fascaplysin **2** (ED_50_ = 0.55 mg/kg) [19], which contrasts with the in vitro results against *S. aureus*, where 3,10-dibromofascaplysin (**5**) was about two orders of magnitude more active against *Staphylococcus* spp. than unsubstituted fascaplysin (**2**). Interestingly, its 9-phenyl derivative **9a** gave similar results in the same sepsis model [19].

#### 2.3.2. Antitumor Efficacy of 3,10-Dibromofascaplysin (5)

Previously, we estimated the antitumor effect of fascaplysin (**2**) against the ascitic form of mouse Ehrlich carcinoma in vivo and demonstrated no reliable increase in life expectancy at a dose of 5 mg/kg [15]. It was also shown earlier that 3,10-dibromofascaplysin had a high therapeutic index for in vitro tests among the series of fascaplysin alkaloids [34]. Therefore, the antitumor effect of this compound was further evaluated in the same model in vivo. The tested substances were administered intraperitoneally (i.p.) to animals at a volume of 0.5 mL daily five times, starting from the next day after tumor inoculation. Compound **5** was administered in a 20% aqueous ethanol solution. Doxorubicin (Dox) was used as a positive control. The daily doses of the test substances were 0.25 mg/kg for Dox and 7.56 mg/kg for compound **5**. The obtained results are presented in Table 2.

This study’s results revealed a moderate antitumor potency of compound **5** at a dose of 7.56 mg/kg (total dose 37.8 mg/kg). Survival in this group was 40%, with a 206% increase in lifespan. This antitumor effect was less pronounced to the reference drug (Dox) at a dose of 0.25 mg/kg (total dose 1.25 mg/kg), which resulted in 60% survival and a 284.8% increase in lifespan. The results indicate that modifying the structure of fascaplysin (**2**) can significantly increase its antitumor efficacy.

**Figure 4:**
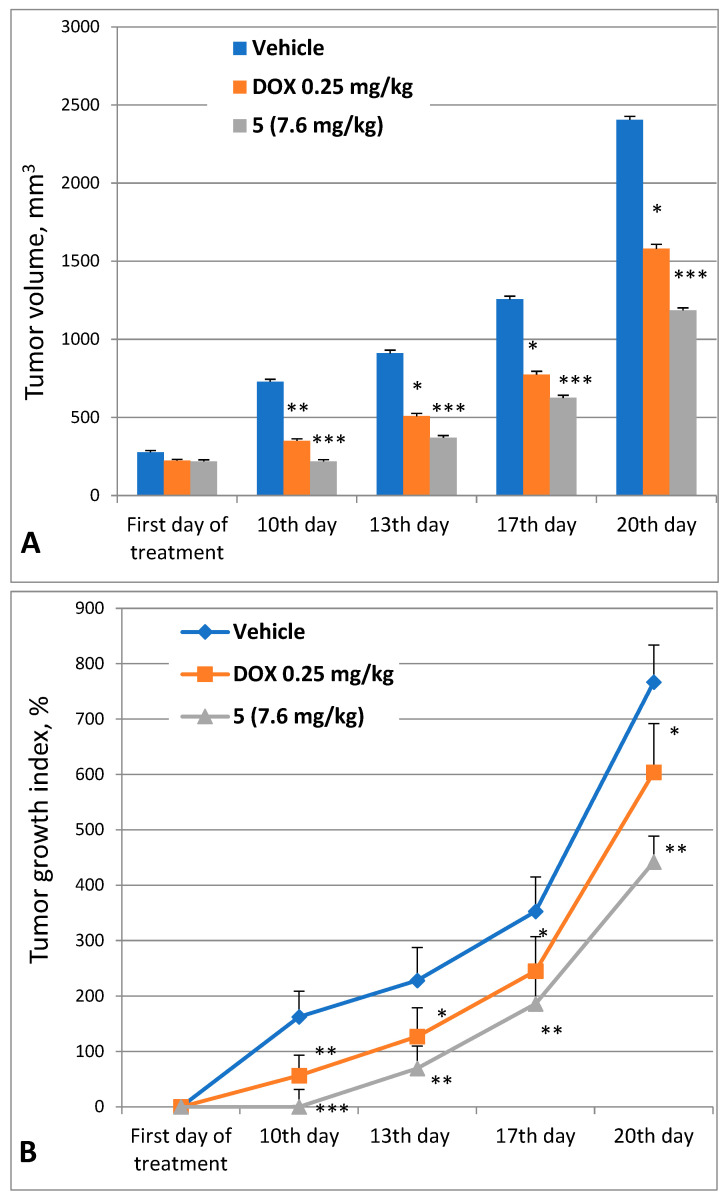
Dynamics of tumor growth. Change in tumor volume (**A**) and tumor growth index (**B**) by day 20 in different groups of animals. Vehicle—control group of animals (untreated); DOX—group of animals treated with doxorubicin at a dose of 0.25 mg/kg; **5**—3,10-dibromofascaplysin at a dose of 7.56 mg/kg. Results are presented as mean ± SEM (*n* = 10). The significance of the differences was estimated via one-way ANOVA, followed by Tukey’s test versus the saline group. Significant differences are presented as * *p* < 0.05, ** *p* < 0.01, and *** *p* < 0.001.

At the same time, 3,10-dibromofascaplysin (**5**) significantly inhibited the growth of solid Ehrlich adenocarcinoma inoculated subcutaneously (Figure 4). The manual measurement of tumor volume revealed rapid tumor growth in the untreated group. The tumor growth indexes on the 10th, 13th, 17th, and 20th days of the experiment in the vehicle group were 162.6%, 228.3%, 352.6%, and 766.9%, respectively. In contrast, the group treated with 3,10-dibromofascaplysin (**5**) showed stagnation in tumor growth. The tumor growth indexes for the 10th, 13th, 17th, and 20th days in the treatment group were 0.1%, 69.5%, 186.2%, and 442.0%, respectively. Thus, on the 10th and 13th days, the tumor volume and growth index in the treatment group were three times lower, and on the 17th and 20th days, they were two times lower than in the vehicle group.

The inhibitory effect of 3,10-dibromofascaplysin on tumor growth was consistent throughout the entire observation period. On the 20th day of the experiment, tumor volume and weight were reduced by half in the group treated with compound **5** compared to the control vehicle group, resulting in a tumor growth inhibition of 49.2%. Meanwhile, in the group treated with a course of Dox, the tumor volume and growth rate were reduced by half on the last day of treatment and by 1.5 times by the final day of the experiment, relative to the vehicle group. The inhibition of tumor growth in the Dox group was only 30%. These results clearly distinguish 3,10-dibromofascaplysin (**5**) from the previously identified lead compound **9a**, for which significant tumor growth inhibition was observed during the course of treatment. However, by the last day of the experiment, its antitumor effect was not significantly different from Dox [19].

## 3. Materials and Methods

### 3.1. Chemistry

All starting materials were commercially available. Commercial reagents were used without any purification. The products were isolated via MPLC: Buchi B-688 pump, glass column C-690 (15 × 460 mm, or PP-cartridge (150 × 12 mm) with Silica gel (particle size 0.015–0.040 mm), as well as a Knauer K-2001 UV detector (Knauer, Berlin, Germany). The analytical examples were purified using a Shimadzu HPLC system (model: LC-20AP), Shimadzu, Kyoto, Japan, equipped with a UV detector (model: SPD-20A) using a Supelco C18 (5 µm, 20 × 250 mm) column (Merck, Taufkirchen, Germany) and a MeOH/water (20:80, 50:50, 70:30) mobile phase via isocratic elution at a flow rate of 15 mL/min. NMR spectra were recorded with an NMR instrument (Bruker, Billerica, MA, USA) operating at 400 MHz (^1^H) and 100 MHz (^13^C). ^1^H NMR spectra were referenced with TMS as the internal standard, in some cases, to the residual signal of the used solvents. Chemical shifts in the ^13^C NMR spectra were determined relative to the ^13^C signal of the TMS or used solvents. Chemical shifts were given on the *δ* scale (ppm). Coupling constants (*J*) were given in Hz. Multiplicities were indicated as follows: s (singlet), d (doublet), t (triplet), q (quartet), m (multiplet), or br (broadened). The original spectra of the relative compounds can be found in the Supplementary Materials. High-resolution mass spectra (HRMS) were obtained using a time-of-flight (TOF) mass spectrometer (model Agilent TOF 6210, Agilent Technologies, Santa Clara, CA, USA) equipped with an electrospray source at atmospheric pressure ionization (ESI).

#### 3.1.1. Preparation of Mixture of Tryptamines **10b** and **17**

A mixture of 4-bromobutanal (1.33 g, 8.8 mmol), 3-bromophenylhydrazine hydrochloride (0.50 g, 2.2 mmol), EtOH (3 mL), and H_2_O (1 mL) was placed into an autoclave and heated at 150 °C for 1 h. After cooling, the mixture was poured into H_2_O (200 mL) and extracted with EtOAc (3 × 50 mL). Then, the aqueous solution was treated with NaOH to pH 12 and extracted with CH_2_Cl_2_ (3 × 50 mL). The combined organic layer was washed with brine (2 × 100 mL), dried over Na_2_SO_4_, and evaporated. After flash column chromatography (EtOAc, then EtOH/NH_3_), compounds **10b** and **17** were isolated as a mixture in a ratio of 1:1 (brown oil, 300 mg, 57%).

#### 3.1.2. Preparation of Substituted 1-Benzoyl-β-Carbolines **12a**-**b**, **18a**-**b**, **19**

Corresponding acetophenone (0.458 mmol) and iodine (92 mg, 0.366 mmol) were added to DMSO (2 mL), and the resulting solution was heated at 90 °C for 1 h. After that, tryptamine, its derivative, or their mixture (0.458 mmol) was added to the solution and this solution was stirred at the same temperature for 3–4 h until completion of the reaction (monitored by TLC). Then, the reaction mixture was cooled to room temperature followed by the addition of water (50 mL) and extraction with EtOAc (2 × 25 mL). The extract was washed with 10% Na_2_S_2_O_3_, dried over Na_2_SO_4_, filtered, and evaporated under reduced pressure. The residue was purified by MPLC using benzene or benzene/hexanes as eluent to give the desired product.

For 1-(2′,4′-dibromobenzoyl)-β-carboline (**12a**): yellow solid, 38%. ^1^H NMR (400 MHz, DMSO-*d*_6_): *δ* 12.23 (br. s, 1H, NH), 8.48 (d, *J* = 4.9, 1H, H-3), 8.44 (d, *J* = 4.9, 1H, H-4), 8.34 (d, *J* = 7.9, 1H, H-5), 8.02 (d, *J* = 1.9, 1H, H-3′), 7.85 (d, *J* = 8.0, 1H, H-8), 7.76 (dd, *J* = 8.3, 1.9, 1H, H-5′), 7.64 (ddd, *J* = 7.2, 7.2, 1.0, 1H, H-7), 7.57 (d, *J* = 8.3, 1H, H-6′), 7.35 (ddd, *J* = 7.2, 7.2, 1.0, 1H, H-6). ^13^C NMR (100 MHz, DMSO-*d*_6_): *δ* 195.9, 142.0, 140.5, 137.9, 135.3, 134.8, 134.3, 131.4, 131.0, 130.3, 129.2, 128.3, 123.2, 121.9, 120.5, 120.0, 119.8, 113.1. HRMS-ESI, *m*/*z*: [M + H]^+^ calculated for C_18_H_11_^79^Br_2_N_2_O^+^ 428.9238, found 428.9264.

For 7-bromo-1-(2′,5′-dibromobenzoyl)-β-carboline (**12b**): yellow solid, 47%. ^1^H NMR (400 MHz, CDCl_3_): *δ* 10.45 (br. s, 1 H), 8.57 (d, *J* = 4.9 Hz, 1 H), 8.16 (d, *J* = 4.9 Hz, 1 H), 8.05 (d, *J* = 8.4 Hz, 1 H), 7.88 (d, *J* = 1.9 Hz, 1 H), 7.79 (d, *J* = 1.5 Hz, 1 H), 7.62 (dd, *J* = 8.2, 1.8 Hz, 1 H), 7.50 (dd, *J* = 8.4, 1.7 Hz, 1 H), 7.46 (d, *J* = 8.2 Hz, 1 H). ^13^C NMR (100 MHz, CDCl_3_): *δ* 197.1, 141.9, 139.3, 138.8, 136.9, 135.7, 135.2, 131.3, 131.0, 130.2, 124.9, 124.6, 123.3, 123.0, 120.9, 119.6, 119.3, 115.2. HRMS-ESI, *m*/*z*: [M + H]^+^ calculated for C_18_H_10_^79^Br_3_N_2_O^+^: 506.8340, found 506.8343.

For 7-bromo-1-(2′,4′-dibromobenzoyl)-β-carboline (**18a**): yellow solid, 20%. ^1^H NMR (400 MHz, CDCl_3_): *δ* 10.45 (br. s, 1H), 8.57 (d, *J* = 4.9 Hz, 1H), 8.15 (d, *J* = 4.9 Hz, 1H), 8.05 (d, *J* = 8.3 Hz, 1H), 7.88 (d, *J* = 1.7 Hz, 1H), 7.79 (d, *J* = 1.1 Hz, 1H), 7.62 (dd, *J* = 8.3, 1.7 Hz, 1H), 7.50 (dd, *J* = 8.3, 1.5 Hz, 1H), 7.46 (d, *J* = 8.2 Hz, 1H). ^13^C NMR (100 MHz, CDCl_3_): *δ* 196.7, 141.4, 138.9, 138.4, 136.5, 135.2, 134.7, 130.9, 130.6, 129.8, 124.4, 124.2, 122.9, 122.6, 120.5, 119.2, 118.9, 114.8. HRMS-ESI, *m*/*z*: [M + H]^+^ calculated for C_18_H_10_^79^Br_3_N_2_O^+^: 506.8340, found 506.8345.

For 5-bromo-1-(2′,4′-dibromobenzoyl)-β-carboline (**18b**): yellow solid, 19%. ^1^H NMR (400 MHz, CDCl_3_): *δ* 10.55 (br. s, 1H), 8.81 (d, *J* = 5.1 Hz, 1H), 8.62 (d, *J* = 5.1 Hz, 1H), 7.88 (d, *J* = 1.8 Hz, 1H), 7.63 (dd, *J* = 3.2, 1.4 Hz, 1H), 7.61 (dd, *J* = 2.9, 1.4 Hz, 1H), 7.54–7.58 (m, 1H), 7.51 (d, *J* = 7.8 Hz, 1H), 7.46 (d, *J* = 8.2 Hz, 1H). ^13^C NMR (100 MHz, CDCl_3_): *δ* 197.5, 142.4, 139.4, 139.1, 137.0, 135.9, 135.1, 131.6, 131.3, 130.4, 130.2, 125.4, 125.1, 121.5, 121.2, 120.3, 118.5, 111.3. HRMS-ESI, *m*/*z*: [M + H]^+^ calculated for C_18_H_10_^79^Br_3_N_2_O^+^ 506.8340, found 506.8347.

For 1-(2′,5′-dibromobenzoyl)-β-carboline (**19**): yellow solid, 40%. ^1^H NMR (400 MHz, CDCl_3_): *δ* 10.52 (br. s., 1H), 8.61 (d, *J* = 4.9 Hz, 1H), 8.35 (d, *J* = 1.9 Hz, 1H), 8.16 (d, *J* = 5.0, 1H), 7.76 (dd, *J* = 8.7, 1.9 Hz, 1H), 7.73 (dd, *J* = 8.1, 1.1 Hz, 1H), 7.59 (dd, *J* = 7.4, 1.7 Hz, 1H), 7.56 (d, *J* = 8.6 Hz, 1H), 7.51 (ddd, *J* = 7.5, 1.1 Hz, 1H), 7.43 (ddd, *J* = 7.7, 1.8 Hz, 1H). ^13^C NMR (100 MHz, CDCl_3_): *δ* 198.4, 140.3, 140.0, 139.4, 137.2, 135.8, 133.4, 132.5, 131.6, 131.0, 130.1, 127.1, 124.9, 122.7, 120.3, 119.5, 114.0, 113.8. HRMS-ESI, *m*/*z*: [M + H]^+^ calculated for C_18_H_11_^79^Br_2_N_2_O^+^ 428.9235, found 428.9241.

#### 3.1.3. Preparation of Substituted 1-Benzoyl-β-Carbolines **20a**–**20b**

Substituted β-carboline (0.1165 mmol) was mixed with NBS (41.5 mg, 0.2330 mmol), and acetic acid (3 mL) was added to the mixture. The resulting mixture was heated at 90 °C for 1 h. Then, the reaction mixture was cooled to room temperature followed by the addition of saturated aqueous solution of Na_2_CO_3_ (50 mL) and extraction with EtOAc (2 × 25 mL). The extract was washed with H_2_O, dried over Na_2_SO_4_, filtered, and evaporated under reduced pressure. The residue was purified by MPLC using benzene to give the target compound.

For 6-bromo-1-(2′,4′-dibromobenzoyl)-β-carboline (**20a**): yellow solid, 67%. ^1^H NMR (400 MHz, CDCl_3_): *δ* 10.42 (br. s., 1H), 8.57 (d, *J* = 4.9 Hz, 1H), 8.31 (s, 1H), 8.13 (d, *J* = 5.0 Hz, 1H), 7.87 (s, 1H), 7.73 (d, *J* = 8.7 Hz, 1H), 7.61 (d, *J* = 8.1 Hz, 1H), 7.52 (d, *J* = 8.7 Hz, 1H), 7.44 (d, *J* = 8.2 Hz, 1H). ^13^C NMR (101 MHz, CDCl_3_): *δ* 197.1, 139.7, 139.2, 138.8, 137.0, 135.7, 135.2, 132.4, 131.1, 130.8, 130.2, 124.9, 124.8, 122.5, 121.0, 119.5, 113.9, 113.6. HRMS-ESI, *m*/*z*: [M + H]^+^ calculated for C_18_H_10_^79^Br_3_N_2_O^+^ 506.8340, found 506.8351.

For 6-bromo-1-(2′,5′-dibromobenzoyl)-β-carboline (**20b**): yellow solid, 65%. ^1^H NMR (400 MHz, CDCl_3_): *δ* 10.42 (br. s., 1H), 8.57 (d, *J* = 5.0 Hz, 1H), 8.31 (d, *J* = 1.7 Hz, 1H), 8.13 (d, *J* = 5.0 Hz, 1H), 7.87 (d, *J* = 1.8 Hz, 1H), 7.74 (dd, *J* = 8.2, 1.8 Hz, 1H), 7.61 (dd, *J* = 8.1, 1.8 Hz, 1H), 7.53 (d, *J* = 8.6 Hz, 1H), 7.45 (d, *J* = 8.2 Hz, 1H). ^13^C NMR (101 MHz, CDCl_3_): *δ* 197.1, 166.8, 139.7, 139.1, 138.8, 135.7, 135.2, 132.4, 131.0, 130.8, 130.2, 124.9, 124.7, 122.4, 121.0, 119.5, 113.9, 113.6. HRMS-ESI, *m*/*z*: [M + H]^+^ calculated for C_18_H_10_^79^Br_3_N_2_O^+^ 506.8340, found 506.8346.

#### 3.1.4. Synthesis of Compound **20c**

β-Carboline **19** (50 mg, 0.117 mmol) was added to the saturated solution of chlorine in acetic acid (4 mL). The resulting mixture was stirred at r.t. for 2 h. Then, the reaction mixture was cooled to room temperature, followed by the addition of a saturated aqueous solution of Na_2_CO_3_ (50 mL) and extraction with EtOAc (2 × 25 mL). The extract was washed with H_2_O, dried over Na_2_SO_4_, filtered, and evaporated under reduced pressure. The residue was purified by MPLC using benzene to give compound **20c** (43 mg, 80%) as a yellow solid. ^1^H NMR (400 MHz, CDCl_3_): *δ* 10.42 (br. s., 1H), 8.56 (d, *J* = 5.0 Hz, 1H), 8.16 (d, *J* = 1.7 Hz, 1H), 8.14 (d, *J* = 4.9 Hz, 1H), 7.87 (d, *J* = 1.8 Hz, 1H), 7.56–7.62 (m, 3H), 7.45 (d, *J* = 8.2 Hz, 1H). ^13^C NMR (101 MHz, CDCl_3_): *δ* 197.1, 139.4, 139.1, 138.8, 137.2, 135.7, 135.2, 131.0, 130.9, 130.2, 129.8, 126.7, 124.8, 121.9, 121.7, 121.0, 119.5, 113.2. HRMS-ESI, *m*/*z*: [M + H]^+^ calculated for C_18_H_10_^79^Br_2_^35^ClN_2_O^+^ 462.8848, found 462.8876.

#### 3.1.5. Preparation of Compound **20d**

β-Carboline **19** (50 mg, 0.1165 mmol) was added to a saturated solution of chlorine in acetic acid (4 mL). The resulting mixture was heated at 60 °C for 1 h. Then, the reaction mixture was cooled to room temperature followed by the addition of saturated aqueous solution of Na_2_CO_3_ (50 mL) and extraction with EtOAc (2 × 25 mL). The extract was washed with H_2_O, dried over Na_2_SO_4_, filtered, and evaporated under reduced pressure. The residue was purified by MPLC using benzene to give compound **20d** (37 mg, 64%) as a yellow solid. ^1^H NMR (400 MHz, CDCl_3_): *δ* 10.47 (br. s., 1H), 8.64 (d, *J* = 5.0 Hz, 1H), 8.17 (dd, *J* = 5.0, 1.0 Hz, 1H), 8.10 (d, *J* = 1.8 Hz, 1H), 7.92 (d, *J* = 1.8 Hz, 1H), 7.69 (d, *J* = 1.8 Hz, 1H), 7.66 (dd, *J* = 8.2, 1.8 Hz, 1H), 7.48 (d, *J* = 8.2 Hz, 1H). ^13^C NMR (101 MHz, CDCl_3_): *δ* 196.8, 139.6, 138.5, 137.0, 136.6, 135.8, 135.7, 131.1, 130.2, 128.8, 126.8, 125.0, 122.8, 121.0, 120.2, 119.8, 118.2, 115.9. HRMS-ESI, *m*/*z*: [M + H]^+^ calculated for C_18_H_9_^79^Br_2_^35^Cl_2_N_2_O^+^ 496.8458, found 496.8490.

#### 3.1.6. Preparation of Fascaplysin Derivatives

Substituted 1-benzoyl-β-carboline (0.326 mmol) was heated in a sealed tube at 200–220 °C for 0.5–2 h. After cooling, the reaction mixture was washed with EtOAc (3 × 3 mL) and H_2_O (3 × 10 mL). The combined aqueous layer was acidified with hydrochloric acid and evaporated under reduced pressure to give the target product as a red powder.

For 3-bromofascaplysin (**3**): prepared at 220 °C, 0.5 h, red solid, 84%. ^1^H NMR (400 MHz, CD_3_OD): *δ* 9.35 (d, *J* = 6.2 Hz, 1H, H-6), 8.95 (d, *J* = 6.2 Hz, 1H, H-7), 8.68 (s, 1H, H-4), 8.48 (d, *J* = 8.1 Hz, 1H, H-8), 7.93 (s, 2H, H-1, H-2), 7.88 (t, *J* = 7.6 Hz, 1H, H-10), 7.79 (d, *J* = 8.1 Hz, 1H, H-11), 7.52 (t, *J* = 7.6 Hz, 1H, H-9). ^13^C-NMR (100 MHz, CD_3_OD): *δ* 182.0, 149.4, 148.9, 143.1, 136.0, 135.6, 132.3, 132.2, 127.7, 127.6, 125.1, 124.5, 124.5, 123.8, 121.1, 120.9, 120.3, 114.5. HRMS-ESI, *m*/*z*: [M]^+^ calculated for C_18_H_10_^79^BrN_2_O^+^ 348.9974, found 348.9980.

For 3,10-dibromofascaplysin (**5**): prepared at 220 °C, 0.5 h, red solid, 91%. ^1^H NMR (400 MHz, CD_3_OD): δ 9.38 (d, *J* = 6.3 Hz, 1H), 8.96 (d, *J* = 6.3 Hz, 1H), 8.69 (s, 1H), 8.41 (d, *J* = 8.6 Hz, 1H), 8.04 (d, *J* = 1.6 Hz, 1H), 7.96 (d, *J* = 0.9 Hz, 2H), 7.70 (dd, *J* = 8.6, 1.7 Hz, 1H). ^13^C NMR (100 MHz, CD_3_OD): *δ* 180.2, 147.7, 147.6, 140.8, 134.0, 131.4, 130.7, 128.7, 126.5, 126.3, 126.0, 124.8, 122.7, 119.5, 119.5, 118.7, 118.4, 115.9. ^13^C-NMR (100 MHz, DMSO-*d*_6_): *δ* 181.3, 148.0, 147.8, 140.2, 134.4, 131.2, 130.5, 128.2, 127.7, 127.1, 126.6, 126.1, 123.5, 123.3, 120.8, 119.6, 118.6, 116.4. HRMS-ESI, *m*/*z*: [M]^+^ calculated for C_18_H_9_^79^Br_2_N_2_O^+^ 426.9079, found 426.9085.

For 12,13-dihydro-2,10-dibromo-13-oxopyrido[1,2-*a*:3,4-*b*′]diindol-5-ium chloride (**13**): prepared at 220 °C, 0.5 h, red solid, 75%. ^1^H NMR (400 MHz, CD_3_OD): *δ* 9.40 (d, *J* = 6.2 Hz, 1H), 8.98 (d, *J* = 6.2 Hz, 1H), 8.71 (s, 1H), 8.42 (d, *J* = 8.5 Hz, 1H), 8.03 (d, *J* = 1.3 Hz, 1H), 7.97 (s, 2H), 7.70 (dd, *J* = 8.5, 1.3 Hz, 1H). ^13^C NMR (100 MHz, CD_3_OD): *δ* 180.6, 148.1, 148.0, 141.2, 134.5, 131.1, 129.1, 127.0, 126.8, 126.4, 125.2, 123.1, 123.0, 119.9, 119.1, 118.8, 116.3, 116.0. HRMS-ESI, *m*/*z*: [M]^+^ calculated for C_18_H_9_^79^Br_2_N_2_O^+^ 426.9079, found 426.9087.

For 12,13-dihydro-3,8-dibromo-13-oxopyrido[1,2-*a*:3,4-*b*′]diindol-5-ium chloride (**14**): prepared at 220 °C, 0.5 h, red solid, 93%. ^1^H NMR (400 MHz, CD_3_OD): *δ* 9.44 (d, *J* = 4.7 Hz, 1H), 9.36 (d, *J* = 4.5 Hz, 1H), 8.76 (s, 1H), 7.98 (s, 2H), 7.76–7.88 (m, 3H). ^13^C NMR (100 MHz, CD_3_OD): *δ* 180.1, 148.1, 147.5, 140.1, 134.5, 134.2, 131.4, 130.7, 126.8, 126.0, 122.7, 122.4, 120.3, 119.5, 118.9, 118.9, 118.5, 112.2. HRMS-ESI, *m*/*z*: [M]^+^ calculated for C_18_H_9_^79^Br_2_N_2_O^+^ 426.9079, found 426.9083.

For 12,13-dihydro-3,9-dibromo-13-oxopyrido[1,2-*a*:3,4-*b*′]diindol-5-ium chloride (**21**): prepared at 200 °C, 2 h, red solid, 64%. ^1^H NMR (400 MHz, DMSO-*d*_6_): *δ* 13.73 (br. s., 1H), 9.69 (s, 1H), 9.19 (s, 1H), 8.91 (s, 1H), 8.85 (s, 1H), 7.95 (m, 3H), 7.74 (d, *J* = 7.6 Hz, 1H). ^13^C NMR (101 MHz, DMSO-*d*_6_): *δ* 181.6, 148.3, 146.2, 140.0, 139.8, 137.1, 134.8, 131.5, 131.3, 130.9, 127.7, 127.3, 124.2, 123.7, 121.5, 120.0, 116.3, 115.5. HRMS-ESI, *m*/*z*: [M]^+^ calculated for C_18_H_9_^79^Br_2_N_2_O^+^ 426.9079, found 426.9085.

For 12,13-dihydro-2,9-dibromo-13-oxopyrido[1,2-*a*:3,4-*b*′]diindol-5-ium chloride (**22**): prepared at 200 °C, 1 h, red solid, 88%. ^1^H NMR (400 MHz, CD_3_OD): 9.43 (d, *J* = 6.2 Hz, 1H), 9.02 (d, *J* = 6.3 Hz, 1H), 8.76–8.74 (m, 2H), 8.03 (dd, *J* = 8.9, 2.0 Hz, 1H), 7.98 (d, *J* = 1.0 Hz, 3H), 7.78 (d, *J* = 9.0 Hz, 1H). ^13^C NMR (100 MHz, CD_3_OD): *δ* 186.0, 148.3, 142.8, 139.4, 137.4, 136.8, 133.4, 130.3, 128.9, 128.7, 128.6, 126.5, 125.4, 122.7, 121.4, 118.2, 118.1, 117.2. HRMS-ESI, *m*/*z*: [M]^+^ calculated for C_18_H_9_^79^Br_2_N_2_O^+^ 426.9079, found 426.9088.

For 12,13-dihydro-2-bromo-9-chloro-13-oxopyrido[1,2-*a*:3,4-*b*′]diindol-5-ium chloride (**23**): prepared at 200 °C, 1 h, red solid, 66%. ^1^H NMR (400 MHz, CD_3_OD): *δ* 9.41 (d, *J* = 6.3 Hz, 1H), 9.00 (d, *J* = 6.2 Hz, 1H), 8.72 (s, 1H), 8.57 (d, *J* = 2.1 Hz, 1H), 7.97 (s, 2H), 7.89 (dd, *J* = 8.9, 2.0 Hz, 1H), 7.81 (d, *J* = 8.9 Hz, 1H). ^13^C NMR (101 MHz, CD_3_OD): *δ* 182.0, 149.5, 147.3, 142.1, 136.0, 133.9, 132.6, 132.4, 130.2, 128.0, 127.8, 124.7, 124.5, 122.3, 121.9, 120.6, 117.4, 116.1. HRMS-ESI, *m*/*z*: [M]^+^ calculated for C_18_H_9_^79^Br^35^ClN_2_O^+^ 382.9581, found 382.9608.

For 12,13-dihydro-2-bromo-9,11-dichloro-13-oxopyrido[1,2-*a*:3,4-*b*′]diindol-5-ium chloride (**24**): prepared at 200 °C, 1 h, red solid, 47%. ^1^H NMR (400 MHz, CD_3_OD): *δ* 9.52 (d, *J* = 6.3 Hz, 1H), 9.10 (d, *J* = 6.2 Hz, 1H), 8.77 (s, 1H), 8.61 (d, *J* = 2.0 Hz, 1H), 8.05 (d, *J* = 1.8 Hz, 1H), 8.01 (s, 2H). ^13^C NMR (101 MHz, CD_3_OD): *δ* 180.8, 148.7, 143.8, 141.9, 135.6, 134.2, 133.1, 132.0, 129.6, 128.4, 127.3, 123.8, 122.9, 122.5, 122.1, 120.2, 120.1, 116.8. HRMS-ESI, *m*/*z*: [M]^+^ calculated for C_18_H_8_^79^Br^35^Cl_2_N_2_O^+^ 416.9192, found 416.9221.

### 3.2. Biological Assay

#### 3.2.1. MIC Values Determination

The antimicrobial activities of fascaplysin (**2**) and its derivatives **3**–**5**, **13**–**14**, and **21**–**24** were studied in comparison to vancomycin on a wide panel of bacteria (*S. aureus* ATCC 29213, *B. cereus* ATCC 10702, *E. faecalis* ATCC 29212, *E. faecium* 132, *E. faecium* 130, *E. faecalis* 583 (VanR), MRSA 88, MRSA PE3R (resistant to doripenem), *S. epidermidis* 2001 MR, *S. aureus* 21555, *M. smegmatis* ATCC 607, and *E. coli* ATCC 25922). The minimum inhibitory concentration (MIC) for the test compounds was determined via the broth microdilution method according to the CLSI guidelines [51], and vancomycin and rifampicin were used as the controls. The reproducibility of the results of three independent repetitions did not extend beyond one dilution, which is acceptable for this method.

#### 3.2.2. In Vivo Efficiency Study of Antibacterial Activity

This animal study was performed in accordance with the European Convention for the Protection of Vertebrate Animals, Directive 86/609/EEC [52], the European Convention for Humane Methods for Animal Welfare and Maintenance [53], and the National Standard of the Russian Federation 33044-2014 “Good Laboratory Practice” [54]. The ethical aspects of the animal experimentation were reviewed and approved by the local ethics committee of the Gause Institute of New Antibiotics, with protocol number 03/2021, dated 12 March 2021.

A comparative study of the efficacy of 3,10-dibromofascaplysin (**5**) and vancomycin was carried out in a mouse staphylococcal sepsis model. Healthy female mice of the SHK colony weighing 18–20 g after a two-week quarantine period were randomized into groups (*n* = 10) and received *S. aureus* (strain 10, clinical isolate, adapted for growth in vivo via five-fold passaging in mice) as the infectious agent. In the experiment, female mice of the SHK colony weighing 18–20 g were used. Initially, the lethal dose (LD_100_) of staphylococcus was determined for this mouse line using the intravenous infection route. The mice deaths were counted daily for 14 days. Thus, the lethal dose (LD_100_) was defined as 8 × 10^8^ CFU/mouse. Afterwards, the mice were seated in cages of 10 heads and infected intravenously with *S. aureus* at a lethal dose, and the efficacy of the tested drugs was determined by the ED_50_ value (i.e., the dose at which 50% of the experimental animals survive). Then, 30 min after infection, the mice were injected intravenously with 3,10-dibromofascaplysin (**5**) at single doses ranging from 0.1 to 4.0 mg/kg or vancomycin at doses ranging from 2.5 to 7.5 mg/kg. 3,10-Dibromofascaplysin (**5**) was dissolved in a mixture pre-warmed to 50–60 °C (0.4 mL, 4:1) of the isotonic solution glucose (ISG) and PEG600, and then the solution was cooled down to room temperature and injected i.p. into the animals. Vancomycin was dissolved in ISG (0.2–0.4 mL). Mice in the control group were i.p. treated by ISG—PEG600 solution (0.4 mL). As a control dose, a group of untreated animals infected with a lethal dose of *S. aureus* was present in the experiment. The animals were observed for 30 days, and the deaths were counted daily. For data analysis and graphing used software XLSTAT 2016, determination of the ED_50_ of the tested compounds was carried out in one experiment under a single control using the Behrens method [55].

#### 3.2.3. In Vivo Study of Antitumor Activity

This study was conducted on mature white female CD-1 (total 145 animals) albino mice weighing 30 ± 3 g at 8–10 weeks of age. The animals were kept under standard conditions in accordance with the rules of SP 2.2.1.3218-14 on the device, equipment, and maintenance of experimental biological vivariums and GOST 33216-2014 “Guidelines for the maintenance and care of animals” [56]. The animals were constantly in the controlled environmental optimal parameters: the temperature was 22 ± 3 °C, the humidity was 50%, and there was a 12 h lighting cycle. Mice were kept in the following conditions: plastic cages, on a bed of small wood shavings, three individuals per 42 × 25 × 14 cm (length × height × width) cage, and in an open mode. The mice had constant access to balanced Delta feeds laboratory animal feed and filtered water. All experimental work with animals was carried out in accordance with the European Directive 2010/63/EC “On the protection of animals used for scientific purposes” [53] and the rules of GOST 33044-2014 “Principles of good laboratory practice” [54]. Work with animals was approved by the local ethics committee of the PIBOC FEB RAS, No 07/2021, dated 8 November 2021.

#### 3.2.4. A Murine Model of Ascite Ehrlich Adenocarcinoma

The Ehrlich mice breast cancer cells was used for ascite and solid tumor inoculation. A murine model of ascite Ehrlich adenocarcinoma was reproduced by an intraperitoneal injection of 3 × 10^6^ cancer cells. 3,10-dibromofascaplysin was administered to animals intraperitoneally (i.p.) at a daily dose of 7.56 mg/kg in a volume of 0.5 mL in a 20% aqueous ethanol solution daily five times, starting from the next day after tumor inoculation. Doxorubicin (Dox) (Veropharm, Russia), an anthracycline antibiotic, was used as a positive control. For the evaluation of the antitumor activity of 3,10-dibromofascaplysin on a murine ascitic Ehrlich adenocarcinoma, the animals were divided into the following groups (*n* = 10): vehicle—0.5 mL of 20% aqueous ethanol solution was administered i.p. daily five times; Dox—0.5 mL of aqueous solutions at a dose of 0.25 mg/kg was administered i.p. daily five times (reference drug—positive control); compound **5**—therapy of 3,10-dibromofascaplysin at a dose of 7.56 mg/kg in 0.5 mL of 20% aqueous ethanol solution was administered i.p. daily five times. The antitumor effect was evaluated by life expectancy and survival of each mice group.

#### 3.2.5. A Murine Model of Solid Ehrlich Adenocarcinoma

Mice were inoculated with 0.2 mL containing 1 × 10^6^ viable EACCs in the right hind limb (thigh) subcutaneously. The animals were randomized and divided into three groups (*n* = 10): vehicle—20% aqueous ethanol solution (negative control); DOX—aqueous solutions 0.25 mg/kg; compound **5**—3,10-dibromofascaplysin in 20% water–ethanol solution 7.56 mg/kg. The treatment started when the primary tumor reached a size of 207–277 mm^3^. 3,10-dibromofascaplysin, Dox, and the vehicle were injected i.p. with 0.5 mL for 5 days (one injection a day). Tumor volume was measured from the 6th day of tumor induction, and then every 4 days for a period of 20 days. Tumor growth was assessed by measuring the volume of the solid tumor using a digital caliper, and it was calculated using the following formula:

$$V=\frac{\pi }{6}\times L\times W\times H$$

where V, tumor volume; L, tumor length; W, tumor width; and H, tumor height.

The evaluation of the antitumor efficacy of compound 5 was carried out using the tumor growth index in each tumor-bearing group to determine the growth rate as a percentage of the value of a tumor over time. The tumor growth index was calculated using the following formula:

$$TumorGrowthIndex\left(\%\right)=\left[\frac{Vc-Vi}{Vi}\right]\times 100$$

where V_c_ is the tumor volume on days 10, 13, 17, and 20 after tumor inoculation, and V_i_ is the tumor volume on the 1st treated day.

On the termination day (20th day of tumor induction), the experimental animals were euthanized via carbon dioxide inhalation, and the tumor mass was removed. To assess the effectiveness of antitumor therapy using the studied drugs, the tumor growth inhibition (TGI, in %) in each treated group of animals was measured. TGI was calculated as described in [57] using the following formula:

$$TGI=\left(1-\frac{Te}{Tc}\right)\times 100$$

where T_e_ means the weight of the treated tumors, and T_c_ means the weight of the tumors in the negative control group of the animals.

### 3.3. Statistical Calculation

Data were shown as mean ± SEM, and *p* < 0.05 was regarded as statistically significant. A one-way analysis of variance (ANOVA), followed by Tukey’s post hoc test, was performed to determine statistical significance using data analysis and graphing software OriginPro 8.5.

## 4. Conclusions

In this study, the antimicrobial activities of synthetic analogs of marine alkaloids 3-bromofascaplysin (**3**), 10-bromofascaplysin (**4**), and 3,10-dibromofascaplysin (**5**) were investigated. Compared with previously obtained data, it was shown for the first time that a significant increase in the antimicrobial activity of fascaplysin occurs not only with the introduction of a substituent at C-9 of cycle A but also with the introduction of a bromine atom at C-3 of cycle E from the opposite side of the scaffold. These findings highlight the need to enhance research in the field of the synthesis and evaluation of the antimicrobial activity of an extended series of monosubstituted fascaplysin derivatives according to this cycle. The introduction of an additional bromine atom at C-10 of 3-bromofascaplysin did not result in a strong enhancement of the antimicrobial properties. However, the study of a series of isomers of compound **5** at positions 2, 8, and 9 revealed that a combination of halogen atoms at C-2 and C-9 increases the antimicrobial activity up to 16 times depending on the strain studied. Unfortunately, a disadvantage of halogenated fascaplysin derivatives **13**, **14**, and **21**–**23** is their low aqueous solubility, which significantly complicates the evaluation of their efficiency on in vivo models.

Sufficient water solubility and comparably low cytotoxicity for normal cells [50] determined the choice of 3,10-dibromofascaplysin for the evaluation of antibacterial and antitumor potential in vivo. In a model of acute bacterial sepsis, we observed that compound **5** has an ED_50_ value of seven times lower that for the reference antibiotic vancomycin. However, in vivo, the compound exhibits comparable efficacy compared to the unsubstituted fascaplysin (**2**), which was significantly less active than compound **5** in vitro. Previously, in a similar experiment, 9-phenylfascaplysin (**9a**) demonstrated similar efficacy [19]. At the same time, testing of the antitumor activity of 3,10-dibromofascaplysin against Ehrlich adenocarcinoma inoculated subcutaneously revealed its higher efficacy than 9-phenylfascaplysin (**9a**). In the case of compound **9a**, a reliable tumor growth inhibition was observed during the treatment course, whereas in the case of compound **5**, the therapeutic effect was significant throughout the whole observation period and achieved 49.2% inhibition of tumor growth on the 20th day after starting the treatment. Therefore, further study of alternative ways to chemically modify fascaplysin is necessary to improve its solubility and chemotherapeutic characteristics. This, in turn, requires additional efforts to develop novel methods for the synthesis and modification of fascaplysin derivatives.

## Figures and Tables

**Table 1:** Antimicrobial activity (MIC *, µg/mL) of fascaplysin (**2**) and its derivatives.

Compound	MIC, µg/mL											
	*S. aureus* ATCC 29213	*B. cereus* ATCC 10702	*E. faecalis* ATCC 29212	*E. faecium* 132	*E. faecium* 130 (VRE)	*E. faecalis* 583 (VRE)	*S. aureus* 88 (MRSA)	*S. aureus* PE3R (MRSA)	*S. epidermidis* 2001 MR	*S. aureus* 21555	*M. smegmatis* ATCC 607	*E. coli* ATCC 25922
Van	0.5	1.0	2.0	0.5	>32.0	32.0	0.5	1.0	1.0	2.0	-	-
Rif	0.018	0.25	-	-	-	-	-	-	-	-	0.03	8.0
**2**	1.0	0.125	8.0	1.0	1.0	≥8.0	1.0	0.5	0.0075	0.03	0.03	8.0
**3**	0.06	0.03	0.25	2.0	1.0	0.5	0.06	0.06	0.03	0.06	0.5	8.0
**4**	0.25	0.13	1.0	8.0	8.0	0.5	0.25	0.25	0.03	0.25	0.5	16.0
**5**	0.06	0.06	0.25	4.0	0.25	0.125	0.06	0.06	0.015	0.03	1.0	>8.0
**13**	0.03	0.015	0.03	0.5	0.06	0.03	0.03	0.015	0.0075	0.015	0.5	>16.0
**14**	0.03	0.015	0.015	0.5	0.015	0.0075	0.015	0.0075	0.0075	0.00375	0.25	4.0
**21**	0.03	0.015	0.015	0.25	0.03	0.015	0.015	0.015	0.0075	0.015	0.25	4.0
**22**	0.015	0.015	0.015	0.25	0.03	0.015	0.015	0.015	0.00375	0.015	0.25	4.0
**23**	0.015	0.015	0.015	0.25	0.03	0.015	0.03	0.015	0.0018	0.015	0.25	>16.0
**24**	0.03	0.03	0.03	0.25	0.015	0.06	0.015	0.015	0.0075	0.015	0.5	>16.0
**9a**	0.03	0.03	0.25	4.0	2.0	0.25	0.015	0.03	0.00375	0.03	0.25	8.0

* Modal values of minimum inhibitory concentration (MIC, µg/mL) of fascaplysin (**2**) and its derivatives determined via the broth microdilution method; vancomycin (Van) and rifampicin (Rif) were used as reference drugs. All experiments were made in three independent repetitions.

**Table 2:** Antitumor efficacy of 3,10-dibromofascaplysin (**5**) and Dox on ascitic Ehrlich adenocarcinoma.

Group	Daily Dose	MST, Days	ILS, %	Survival, %
Control	-	16.1 ± 5.0	-	0
Compound **5**	7.56 mg/kg	49.3 ± 14.63 *	206	40
Dox	0.25 mg/kg	62.0 ± 0.0 **	284.8	60

MST—mean survival time of animals with standard deviation; ILS—increase in life span, %; (-)—negative control (without treatment); Dox—doxorubicin treatment; **5**—3,10-dibromofascaplysin treatment. Results are presented as mean ± SEM, *n* = 10 for each group. The significance of differences was estimated via one-way ANOVA, followed by Tukey’s test versus the saline group. Significant differences are presented as * *p* < 0.05 and ** *p* < 0.01.

## Data Availability

The original data are available from the corresponding author on request.

## Supplementary Materials

The following supporting information can be downloaded via this link: www.mdpi.com/article/10.3390/md23020068/s1. Table S1. Antimicrobial activity of fascaplysin (**2**) and its derivatives in vitro. Table S2. Efficacy (ED_50_ value, mg/kg) of 3,10-dibromofascaplysin (**5**) and vancomycin (Van) on a mouse model of staphylococcal sepsis. Comparison of NMR data of synthetic and natural 3-bromofascaplysin and 3,10-dibromofascaplysin. Spectra data.
