# Supplementary material for: Comparative Evaluation of the Antibacterial and Antitumor Activities of Marine Alkaloid 3,10-Dibromofascaplysin
Source: Mar Drugs. 2025 Feb 6;23(2):68. doi: 10.3390/md23020068 (PMC11857626; doi:10.3390/md23020068)

Supporting Information

Comparative Evaluation of the Antibacterial and Antitumor Activities of Marine Alkaloid 3,10-Dibromofascaplysin

Maxim E. Zhidkov [1,*], Polina A. Smirnova [1], Natalia E. Grammatikova [2], Elena B. Isakova [2], Andrey E. Shchekotikhin [2], Olga N. Styshova [3], Anna A. Klimovich [3] and Aleksandr M. Popov [3]

[1] Department of Chemistry and Materials, Institute of High Technologies and Advanced Materials, FEFU Campus, Far Eastern Federal University, Ajax Bay 10, Russky Island, 690922 Vladivostok, Russia; smirnova_pa@[1]dvfu.ru (P.A.S.)

[2] Laboratory of Chemical Transformation of Antibiotics, Gause Institute of New Antibiotics, 119021 Moscow, Russia; ngrammatikova@yandex.ru (N.E.G.); ebisakova@yandex.ru (E.B.I.); shchekotikhin@mail.ru (A.E.S.)

[3] Departments of Biotechnology and Marine Natural Compounds Chemistry, G.B. Elyakov Pacific Institute of Bioorganic Chemistry, Far Eastern Branch of The Russian Academy of Sciences, 690922 Vladivostok, Russia; krivoshapkoon@mail.ru (O.N.S.); annaklim_1991@mail.ru (A.A.K.); popovam@piboc.dvo.ru (A.M.P.)

* Correspondence: zhidkov.me@dvfu.ru

Contents

Table S1. Antimicrobial activity of fascaplysin (**2**) and its derivatives *in vitro* 3

Table S2. Efficacy (ED_{50} value, mg/kg) of 3,10-dibromofascaplysin (**5**) and vancomycin (Van) on a mouse model of staphylococcal sepsis 4

Comparison of NMR data of synthetic and natural 3-bromofascaplysin and 3,10-dibromofascaplysin 5-8

Spectra Data 9-42

Table S1. Antimicrobial activity of fascaplysin (2) and its derivatives *in vitro*

Compound	MIC, µg/mL											
	***S. aureus* ATCC 29213**	***B. cereus* ATCC 10702**	***E. faecalis* ATCC 29212**	***E. faecium* 132**	***E. faecium* 130 (VRE)**	***E. faecalis* 583 (VRE)**	***S. aureus* 88 (MRSA)**	***S. aureus* PE3R (MRSA)**	***S. epidermidis* 2001 MR**	***S. aureus* 21555**	***M. smegmatis* ATCC 607**	***E. coli* ATCC 25922**
Van	0.5	1.0	2.0	0.5	>32.0	32.0	0.5	1.0	1.0	2.0	-	-
Rif	0.018	0.25	-	-	-	-	-	-	-	-	0.03	8.0
**2**	1.0	0.125	8.0	1.0	1.0	≥8.0	1.0	0.5	0.0075	0.03	0.03	8.0
**3**	0.06	0.03	0.25	2.0	1.0	0.5	0.06	0.06	0.03	0.06	0.5	8.0
**4**	0.25	0.13	1.0	8.0	8.0	0.5	0.25	0.25	0.03	0.25	0.5	16.0
**5**	0.06	0.06	0.25	4.0	0.25	0.125	0.06	0.06	0.015	0.03	1.0	>8.0
**13**	0.03	0.015	0.03	0.5	0.06	0.03	0.03	0.015	0.0075	0.015	0.5	>16.0
**14**	0.03	0.015	0.015	0.5	0.015	0.0075	0.015	0.0075	0.0075	0.00375	0.25	4.0
**21**	0.03	0.015	0.015	0.25	0.03	0.015	0.015	0.015	0.0075	0.015	0.25	4.0
**22**	**0.015**	**0.015**	**0.015**	**0.25**	**0.03**	**0.015**	**0.015**	**0.015**	**0.00375**	**0.015**	**0.25**	**4.0**
**23**	**0.015**	**0.015**	**0.015**	**0.25**	**0.03**	**0.015**	**0.03**	**0.015**	**0.0018**	**0.015**	**0.25**	**>16.0**
**24**	0.03	0.03	0.03	0.25	0.015	0.06	0.015	0.015	0.0075	0.015	0.5	>16.0
**9a**	0.03	0.03	0.25	4.0	2.0	0.25	0.015	0.03	0.00375	0.03	0.25	8.0

Table S2. Efficacy (ED_{50} value, mg/kg) of 3,10-dibromofascaplysin (5) and vancomycin (Van) on a mouse model of staphylococcal sepsis

Compound	Dose, mg/kg	Survival*, %	ED_{50}, mg/kg
3,10-Dibromofascaplysin (**5**)	0.10	20	0.60
	0.25	40	
	0.50	40	
	1.0	60	
	2.0	70	
	4.0	80	
Van	2.5	30	4.2
	3.5	40	
	4.5	50	
	5.5	70	
	6.5	90	
	7.5	100	
Control dose *S. aureus*	-	0	

Comparison of NMR data of synthetic and natural 3-bromofascaplysin and 3,10-dibromofascaplysin

1. 3-Bromofascaplysin (recorded in CD_3OD)[1]

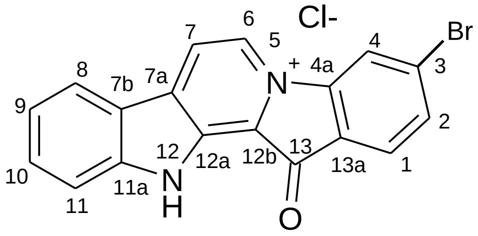

Position	^{1}H-NMR		^{13}C-NMR	
	synthetic	natural	synthetic	natural
1a	-	-	132.2	132.5
1	7.93, s	7.93, s	127.6	127.7
2	7.93, s	7.93, s	135.6	135.8
3	-	-	124.5	124.5
4	8.68, s	8.66, s	120.3	120.4
4a	-	-	149.4	149.5
6	9.35, d (6.2)	9.34, d (6.0)	127.7	127.9
7	8.95, d (6.2)	8.93, d (6.0)	120.9	121.0
7a	-	-	123.8	123.8
7b	-	-	121.1	121.2
8	8.48, d (8.1)	8.45, d (7.5)	125.1	125.3
9	7.52, t (7.6)	7.51, t (7.5)	124.5	124.7
10	7.88, t (7.6)	7.87, t (7.5)	136.0	136.1

[1] NMR data of natural 3-bromofascaplysin presented in Segraves, N. L.; Lopez, S.; Johnson, T. A.; Said, S. A.; Fu, X.; Schmitz, F. J.; Pietraszkiewicz, H.; Valeriotec, F. A.; Crews, P. Structures and cytotoxicities of fascaplysin and related alkaloids from two marine phyla—*Fascaplysinopsis* sponges and *Didemnum* tunicates. *Tetrahedron Lett.* **2003**, *44,* 3471-3475.

11	7.79, d (8.1)	7.76, d (7.5)	114.5	114.7
11a	-	-	148.9	149.0
12a	-	-	132.3	132.3
12b	-	-	143.1	143.2
13	-	-	182.0	182.1

2. 3,10-Dibromofascaplysin[2]

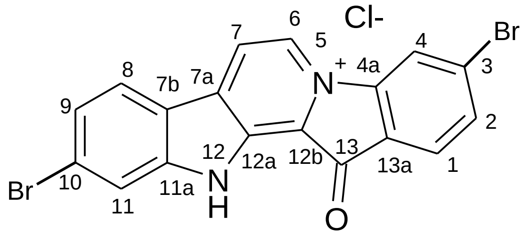

Position	^{1}H-NMR (recorded in CD_3OD)		ЯМР-^{13}C-NMR (recorded in DMSO-d_6)	
	synthetic	natural	synthetic	natural
1a	-	-	130.5	130.6
1	7.96, d (0.9), 2H	7.96, d (1.2)	127.1	127.0
2		7.96, d (1.2)	134.4	134.4
3	-	-	123.3	123.3
4	8.69, s	8.70, t (0.9)	119.6	119.5
4a	-	-	148.0	148.0
6	9.38, d (6.3)	9.39, d (6.4)	127.7	127.6
7	8.96, d (6.3)	8.97, d (6.4)	120.8	120.7
7a	-	-	140.2	140.3
7b	-	-	118.6	118.7
8	8.41, d (8.6)	8.41, d (8.4)	126.1	126.2
9	7.70, dd (8.6, 1.7)	7.70, dd (8.5, 1.6)	126.6	126.5
10	-	-	128.2	128.1
11	8.04, d (1.6)	8.03, d (1.2)	147.8	147.7

[2] NMR data of natural 3,10-dibromofascaplysin presented in Segraves, N. L.; Robinson, S. J.; Garcia, D.; Said, S. A.; Fu, X.; Schmitz, F. J.; Pietraszkiewicz, H.; Valeriote, F. A.; Crews, P. Comparison of fascaplysin and related alkaloids: a study of structures, cytotoxicities, and sources. *J. Nat. Prod.* **2004**, *67*, 783-792.

11a	-	-	116.4	116.5
12a	-	-	131.2	131.3
12b	-	-	123.5	123.6
13	-	-	181.3	181.2

Spectra Data

^{1}H NMR spectra of 1-(2.4-dibromobenzoyl)-β-carboline (12a).

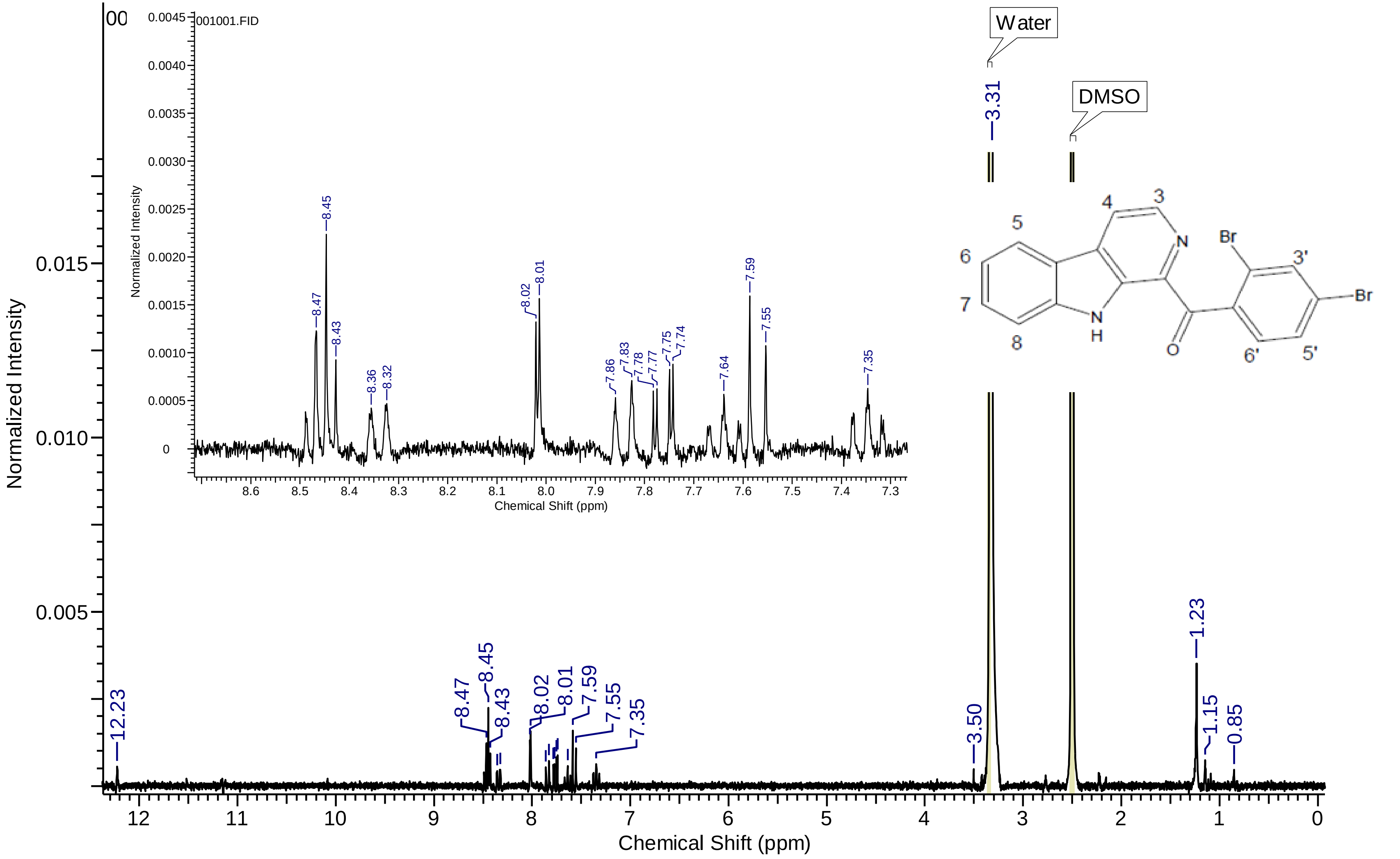

^{13}C NMR spectra of 1-(2.4-dibromobenzoyl)-β-carboline (12a).

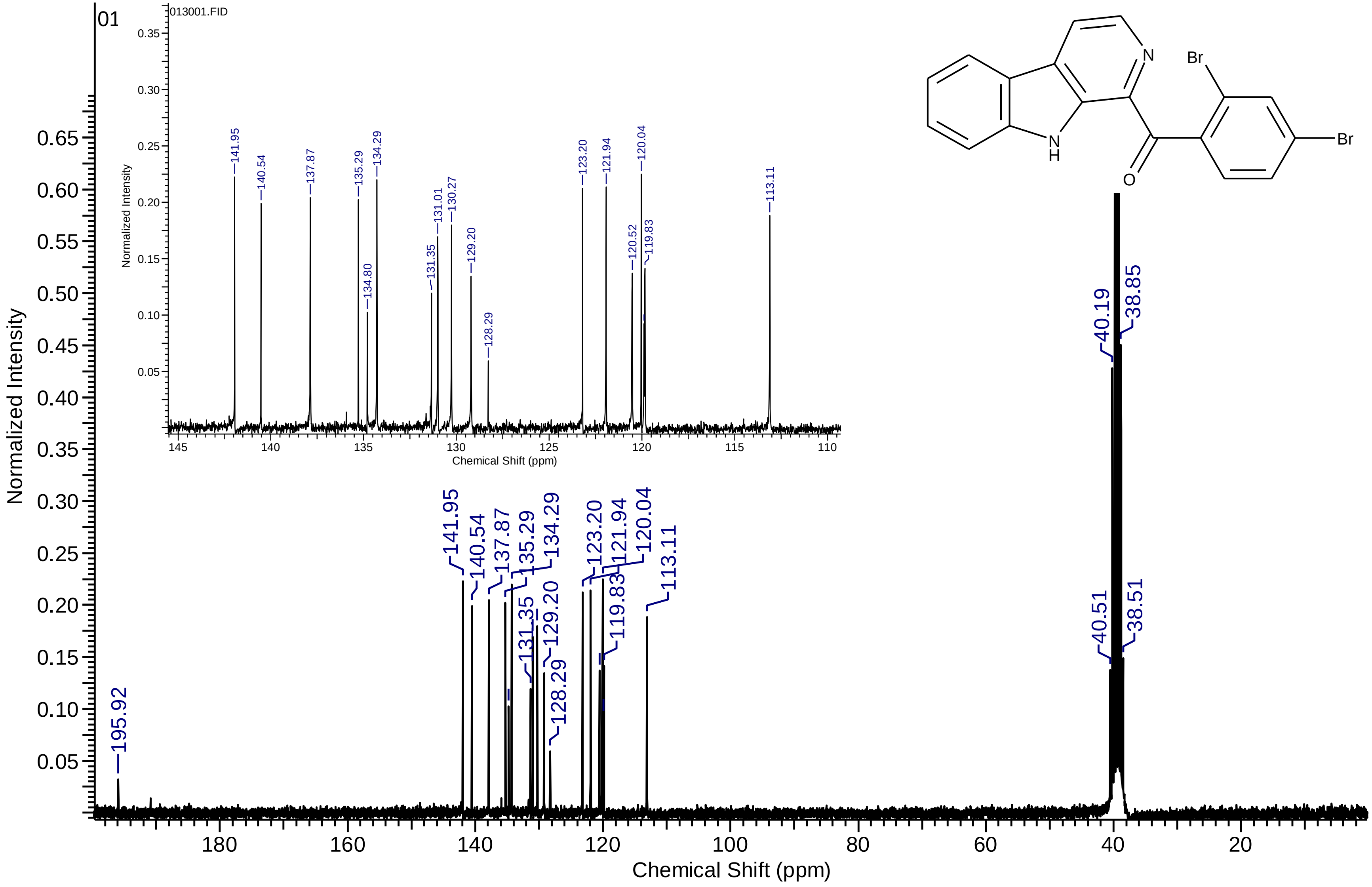

^{1}H NMR spectra of 7-bromo-1-(2′,5′-dibromobenzoyl)-β-carboline (12b).

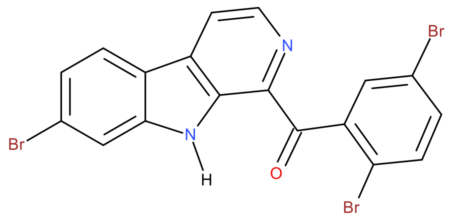

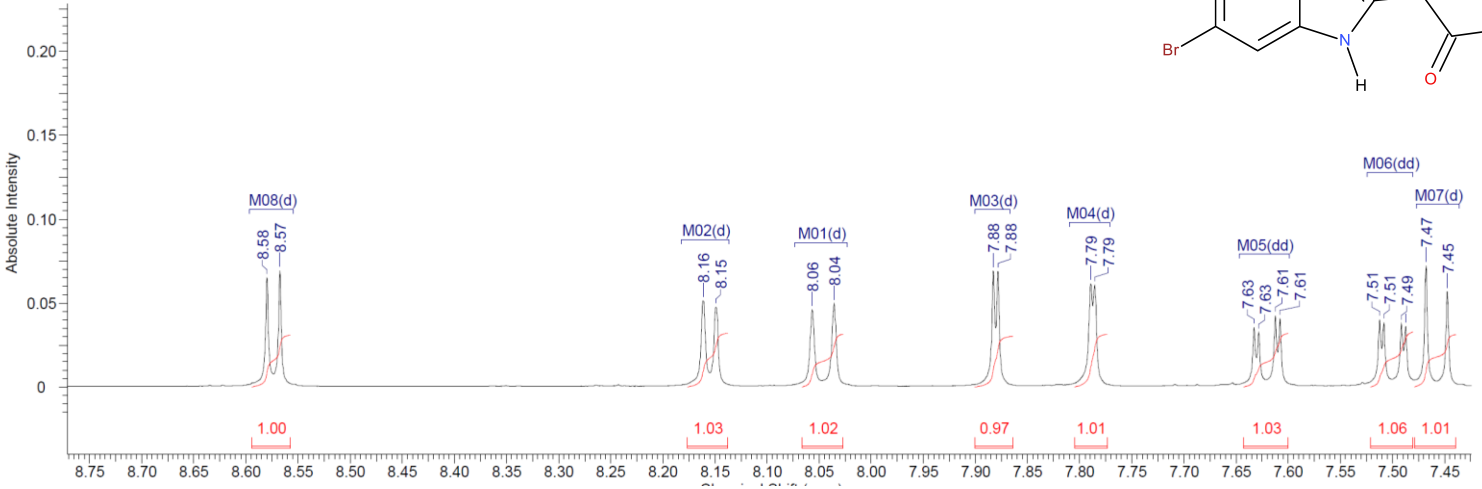

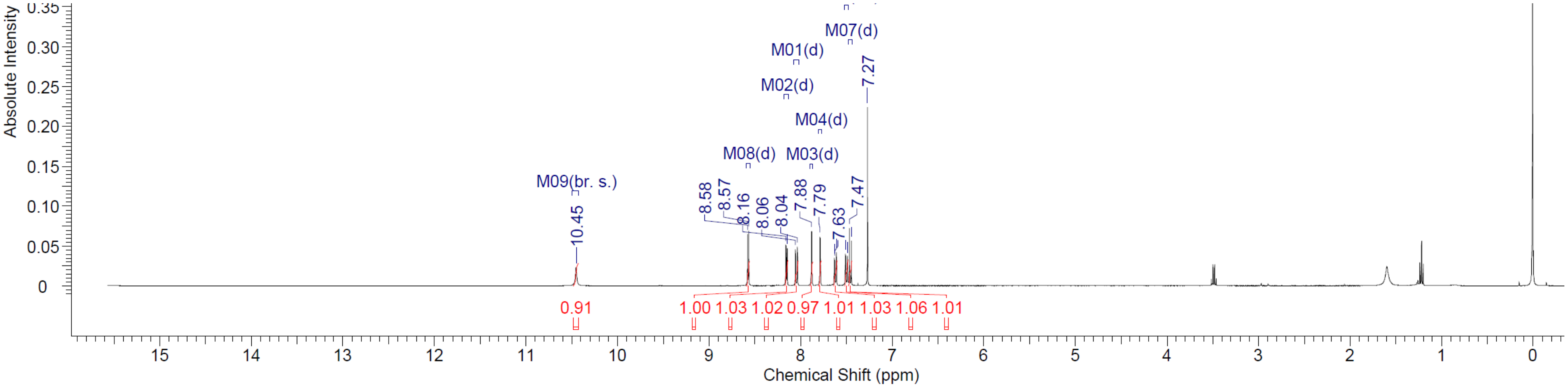

^{13}C NMR spectra of 7-bromo-1-(2′,5′-dibromobenzoyl)-β-carboline (12b).

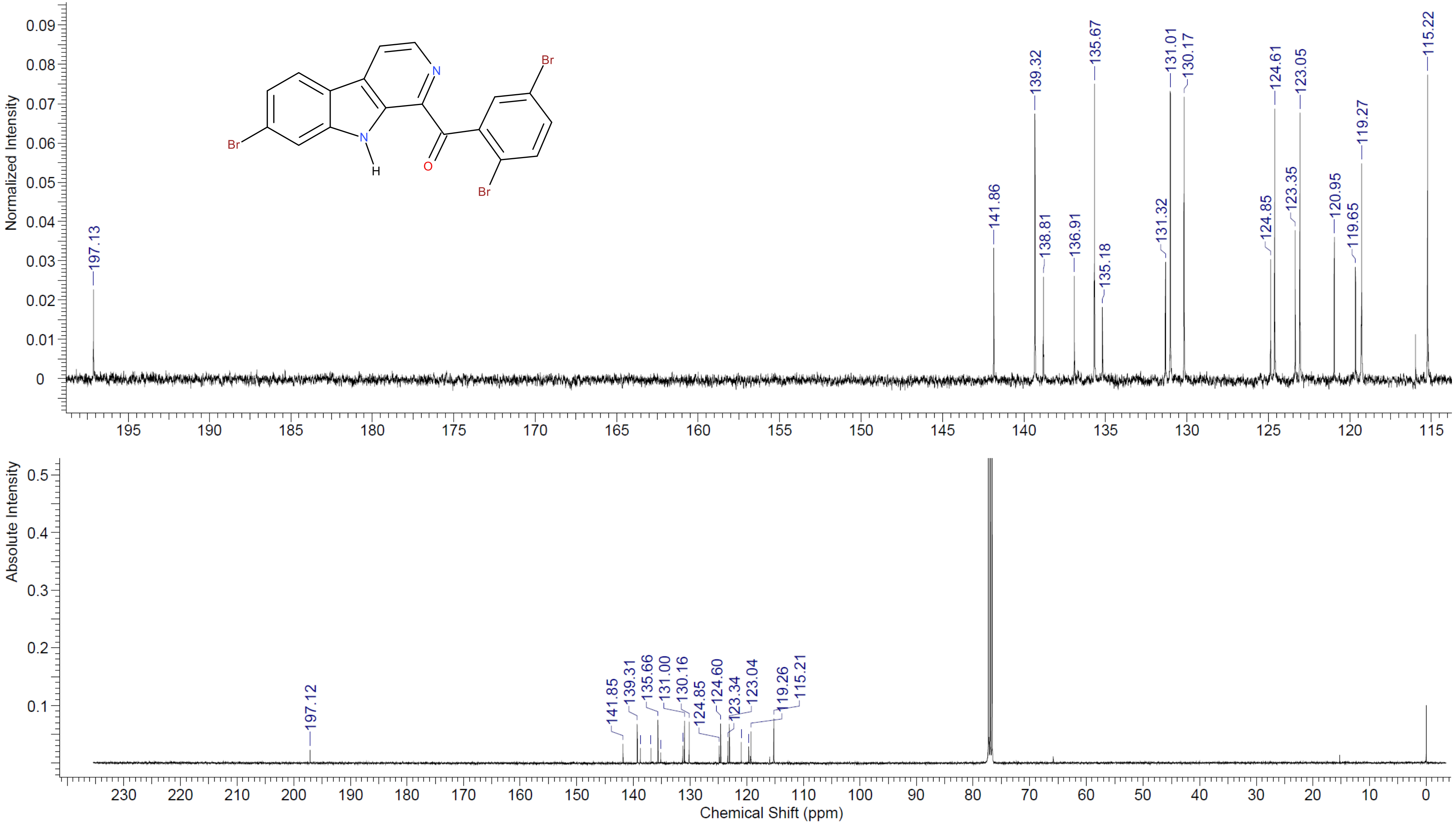

^{1}H NMR spectra of 7-bromo-1-(2′,4′-dibromobenzoyl)-β-carboline (18a).

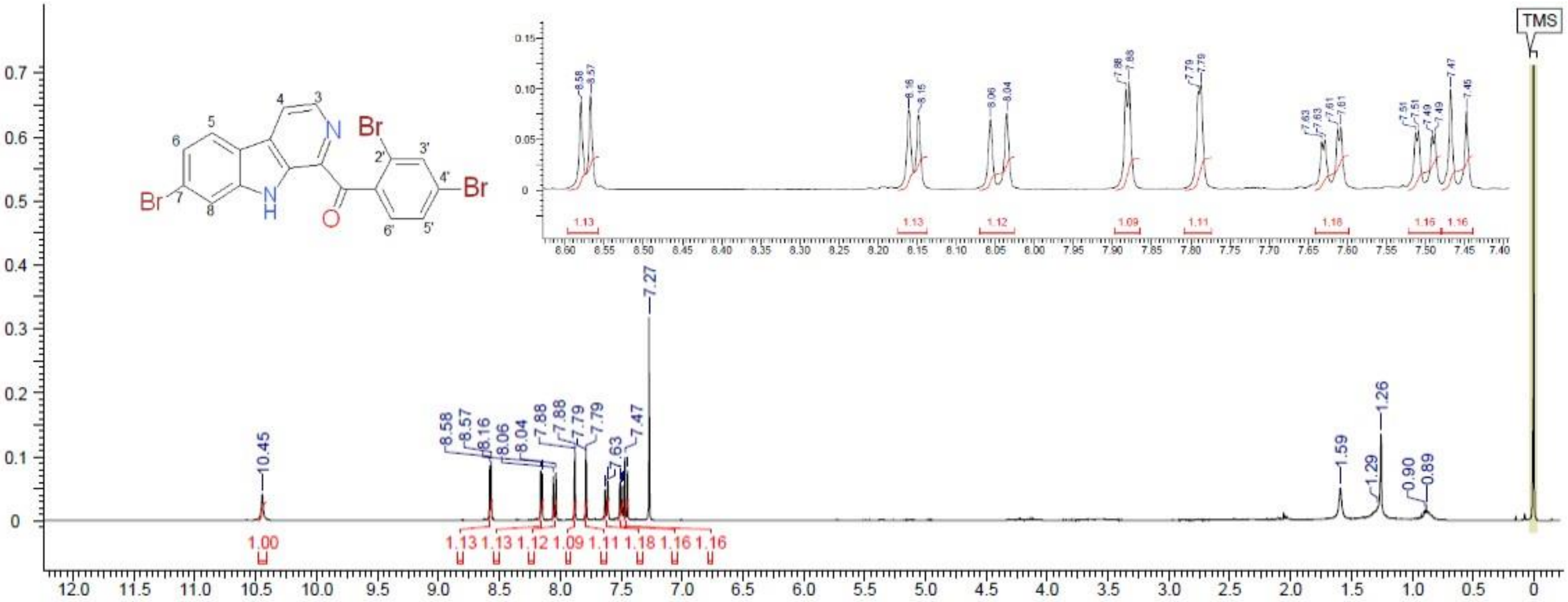

^{13}C NMR 7-bromo-1-(2′,4′-dibromobenzoyl)-β-carboline (18a).

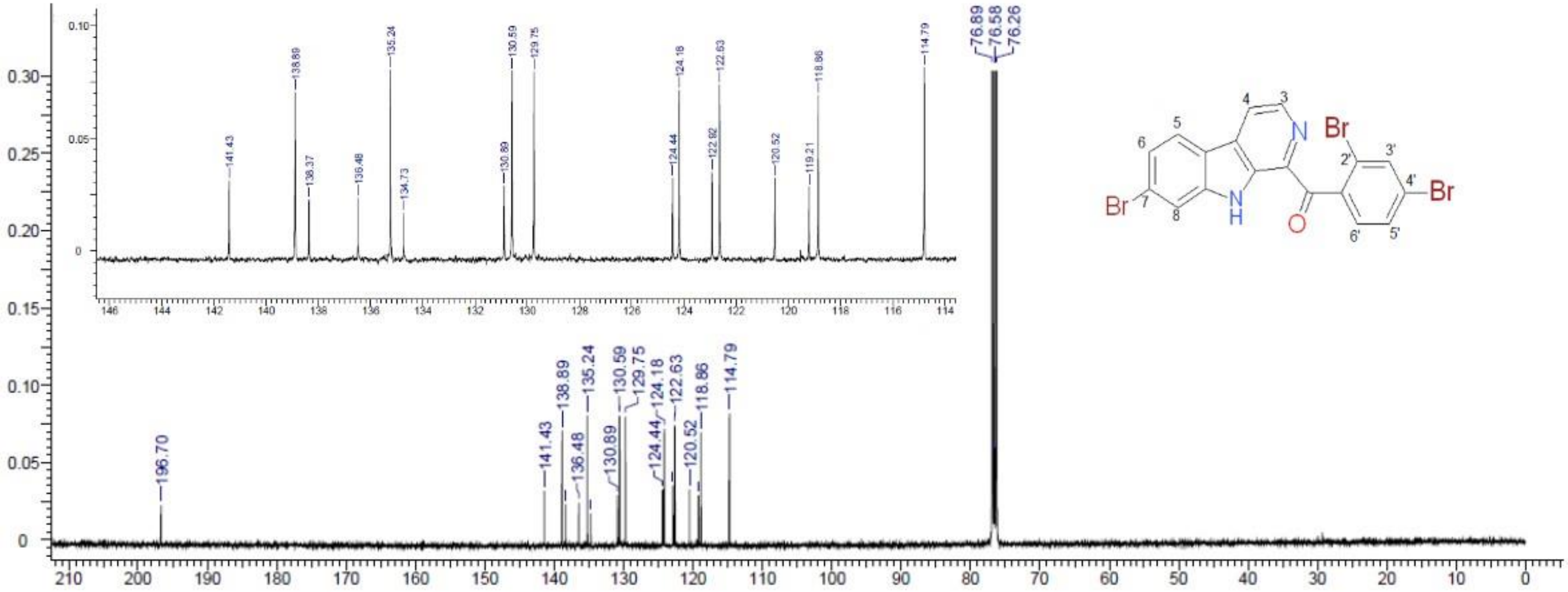

^{1}H NMR spectra of 15-bromo-1-(2′,4′-dibromobenzoyl)-β-carboline (18b).

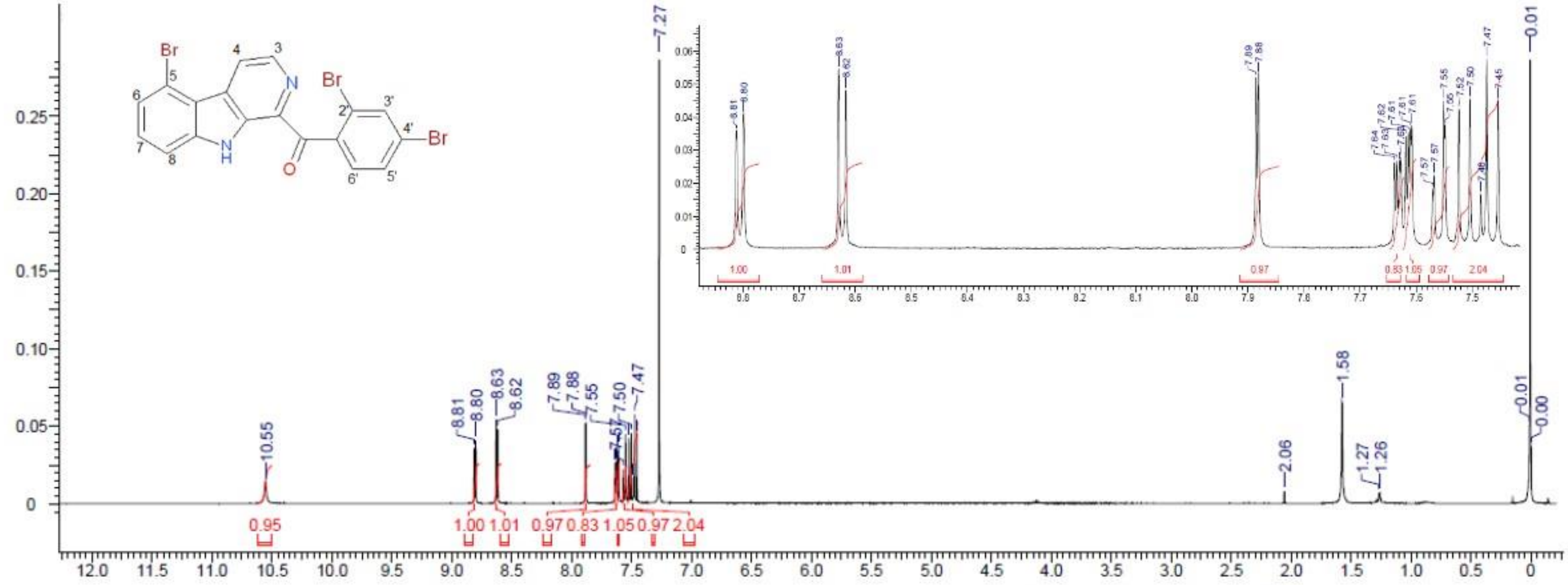

^{13}C NMR spectra of 5-bromo-1-(2′,4′-dibromobenzoyl)-β-carboline (18b).

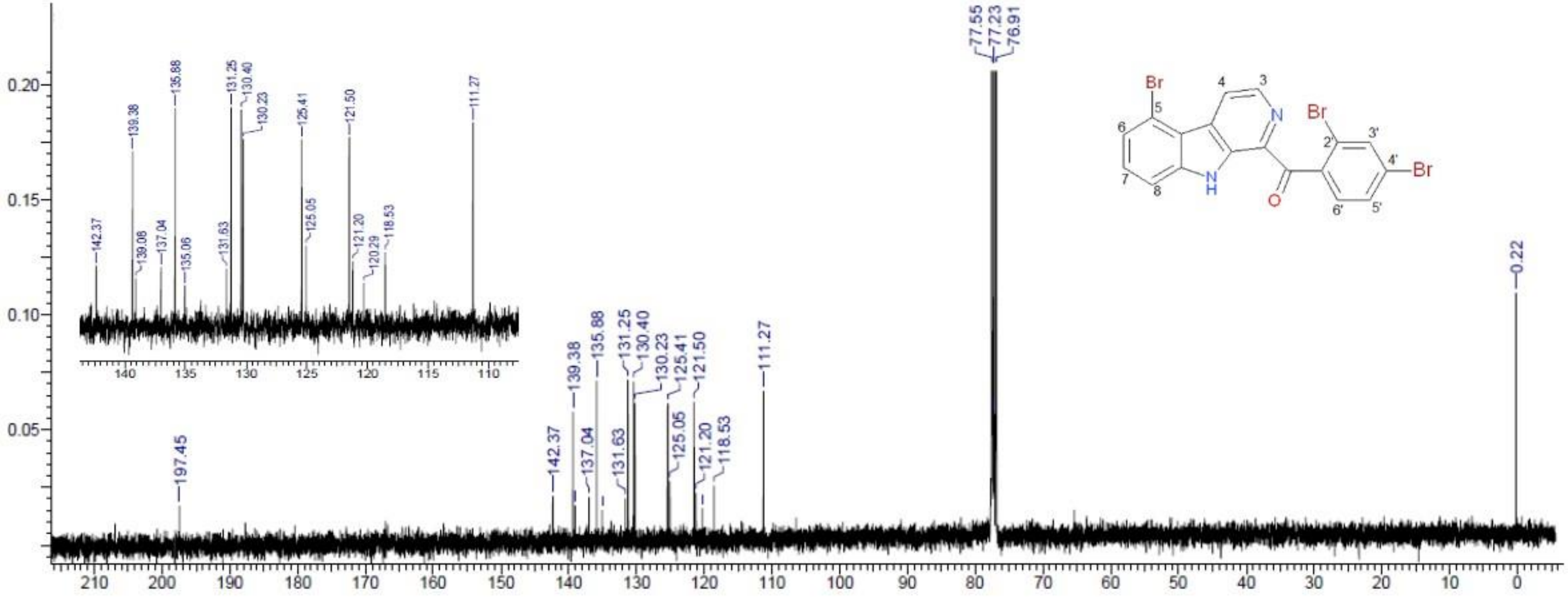

^{1}H NMR spectra of 1-(2',5'-dibromobenzoyl)-β-carboline (19).

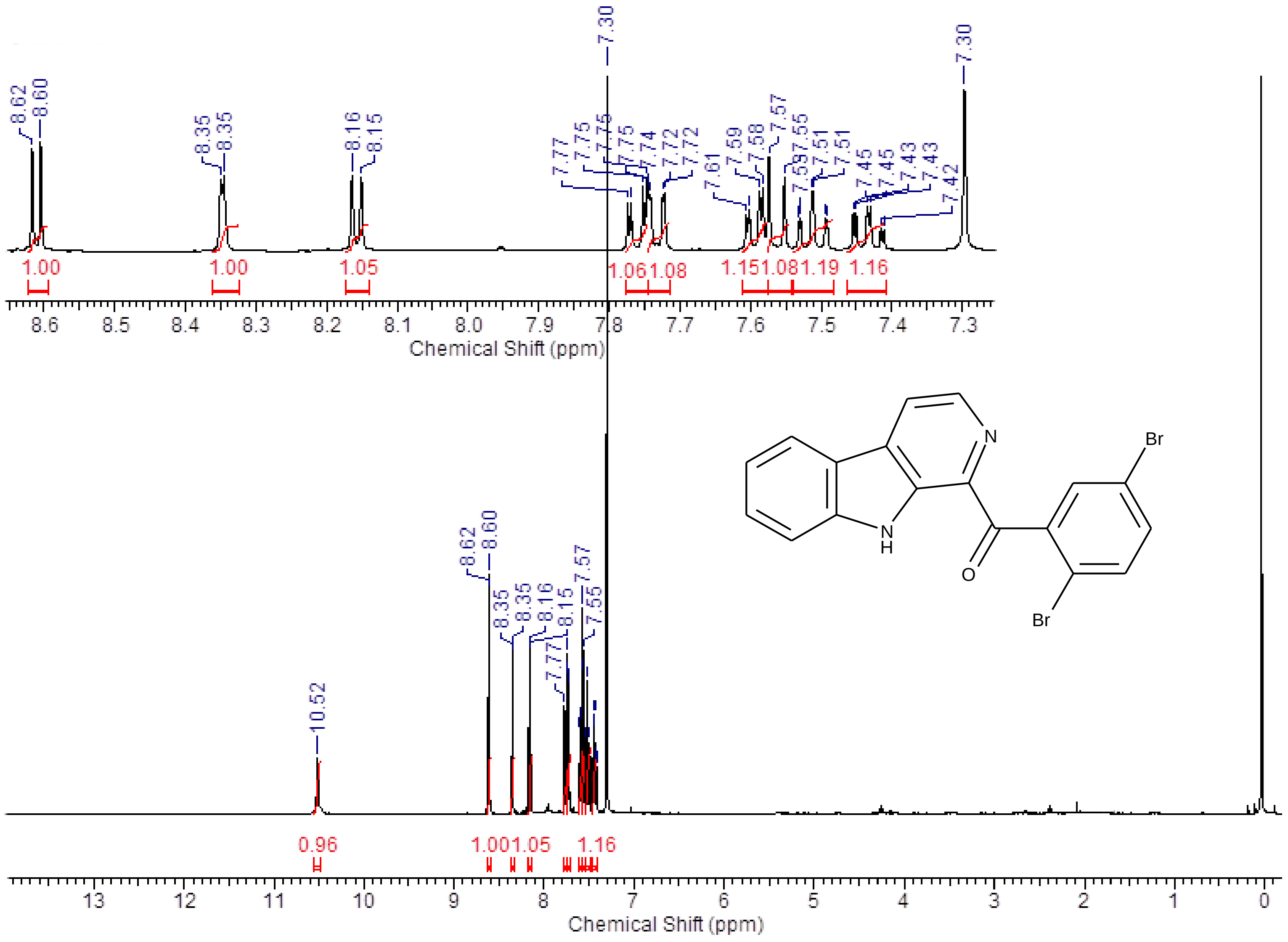

^{13}C NMR spectra of 1-(2’,5’-dibromobenzoyl)-β-carboline (19).

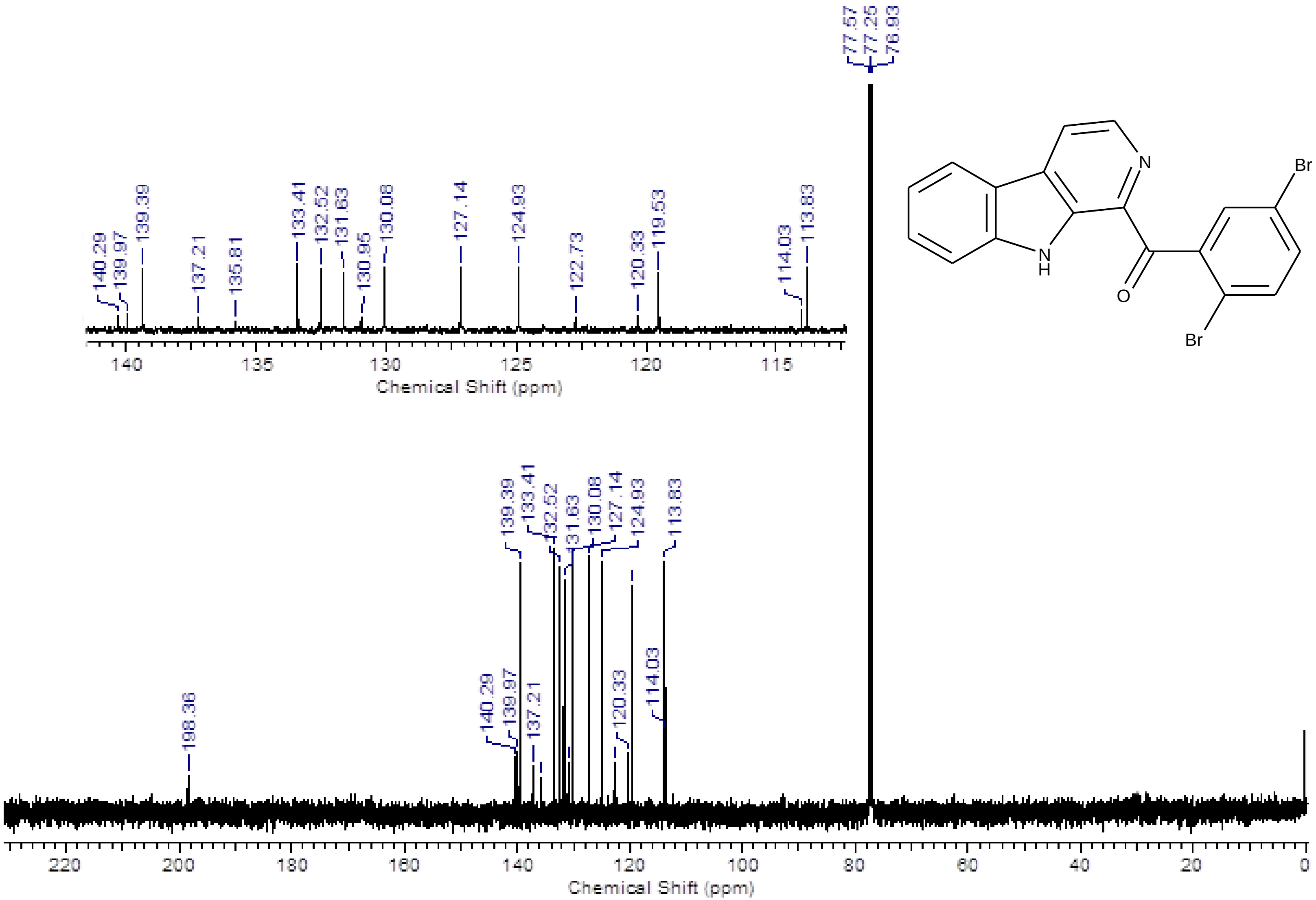

^{1}H NMR spectra of 6-bromo-1-(2’,4’-dibromobenzoyl)-β-carboline (20a).

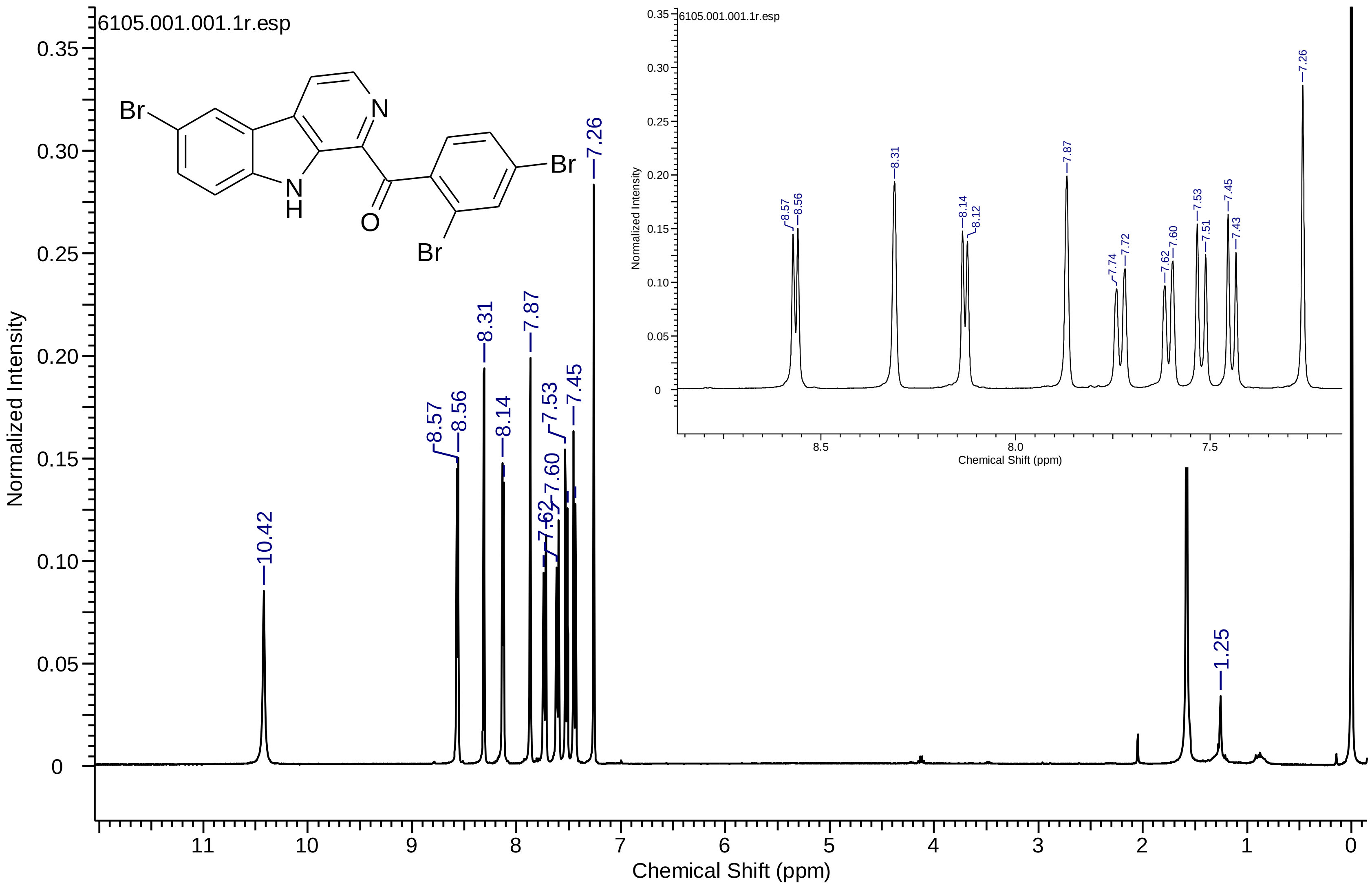

^{13}C NMR spectra of 6-bromo-1-(2',4'-dibromobenzoyl)-β-carboline (20a).

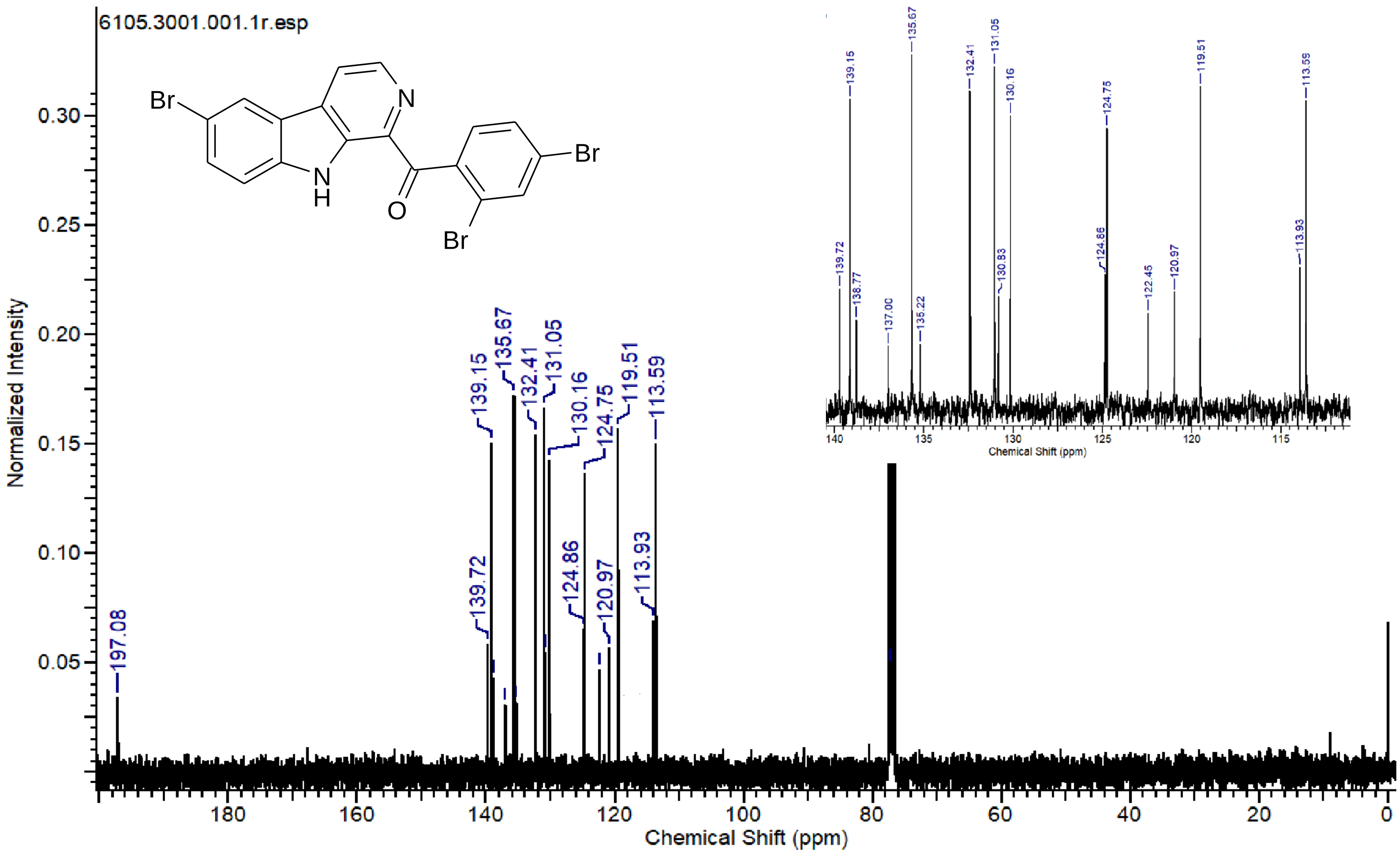

^{1}H NMR spectra of 6-bromo-1-(2’,5’-dibromobenzoyl)-β-carboline (20b).

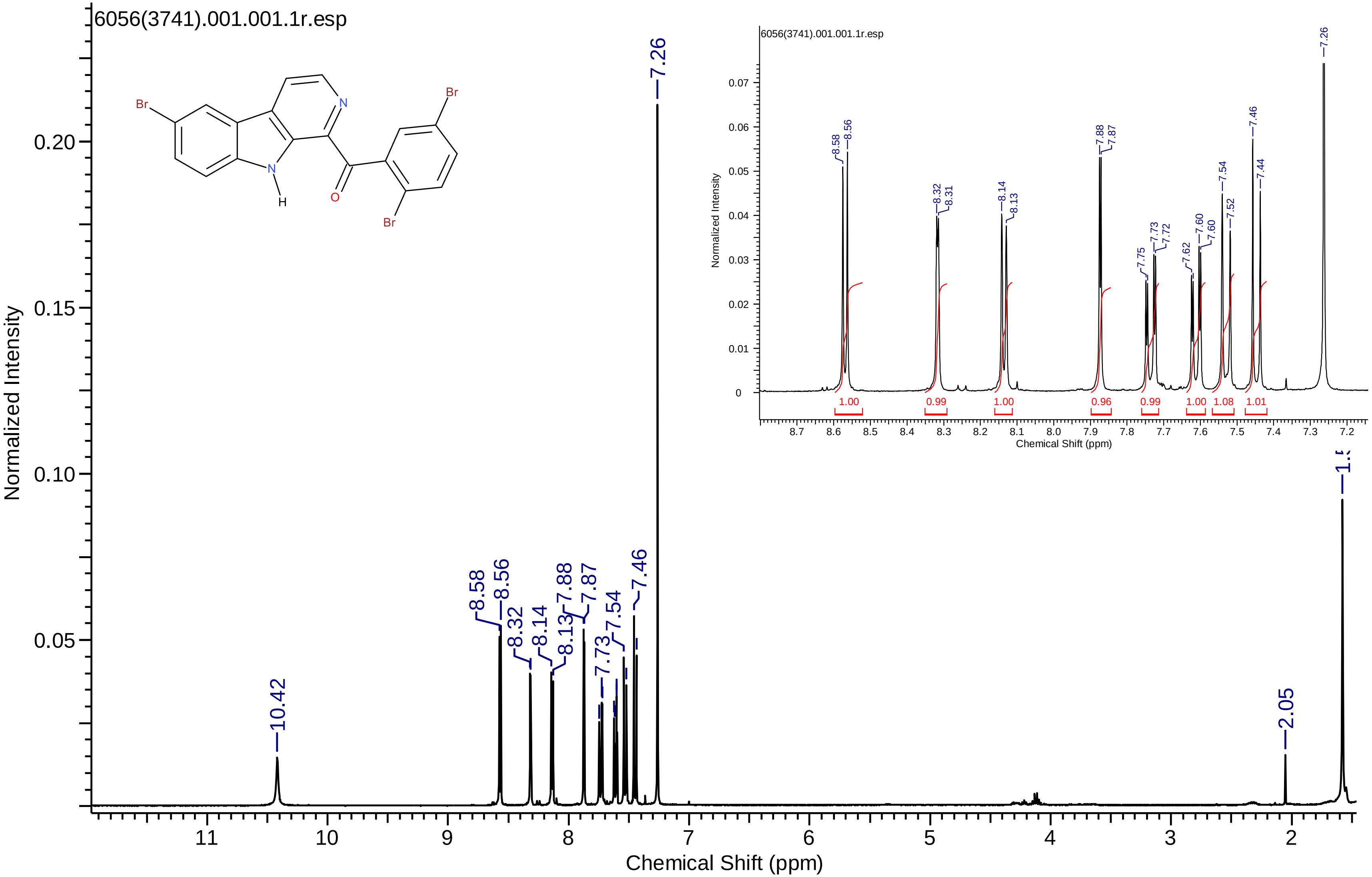

^{13}C NMR spectra of 6-bromo-1-(2’,5’-dibromobenzoyl)-β-carboline (20b).

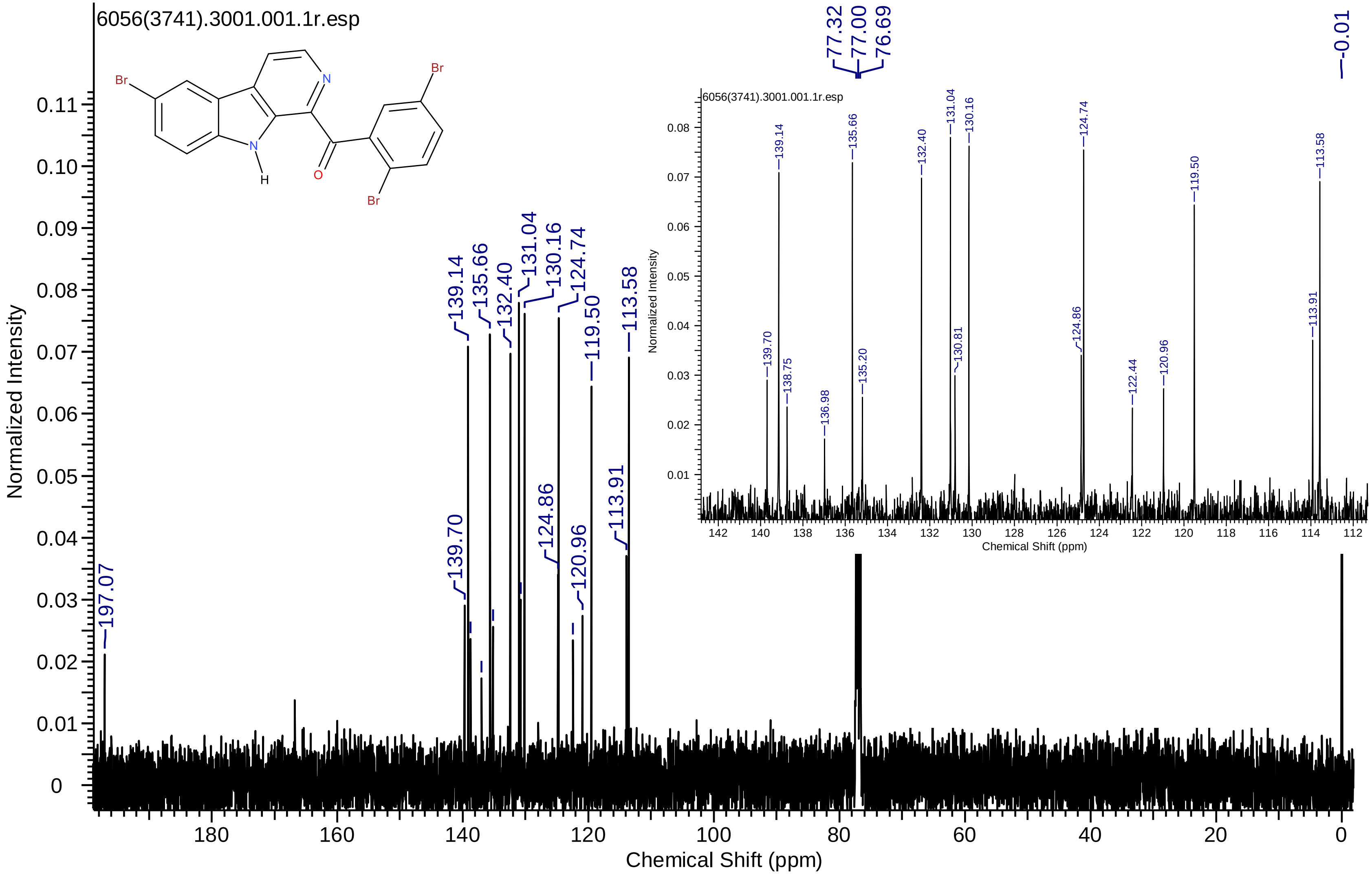

^{1}H NMR spectra of 6-chloro-1-(2’,5’-dibromobenzoyl)-β-carboline (20c).

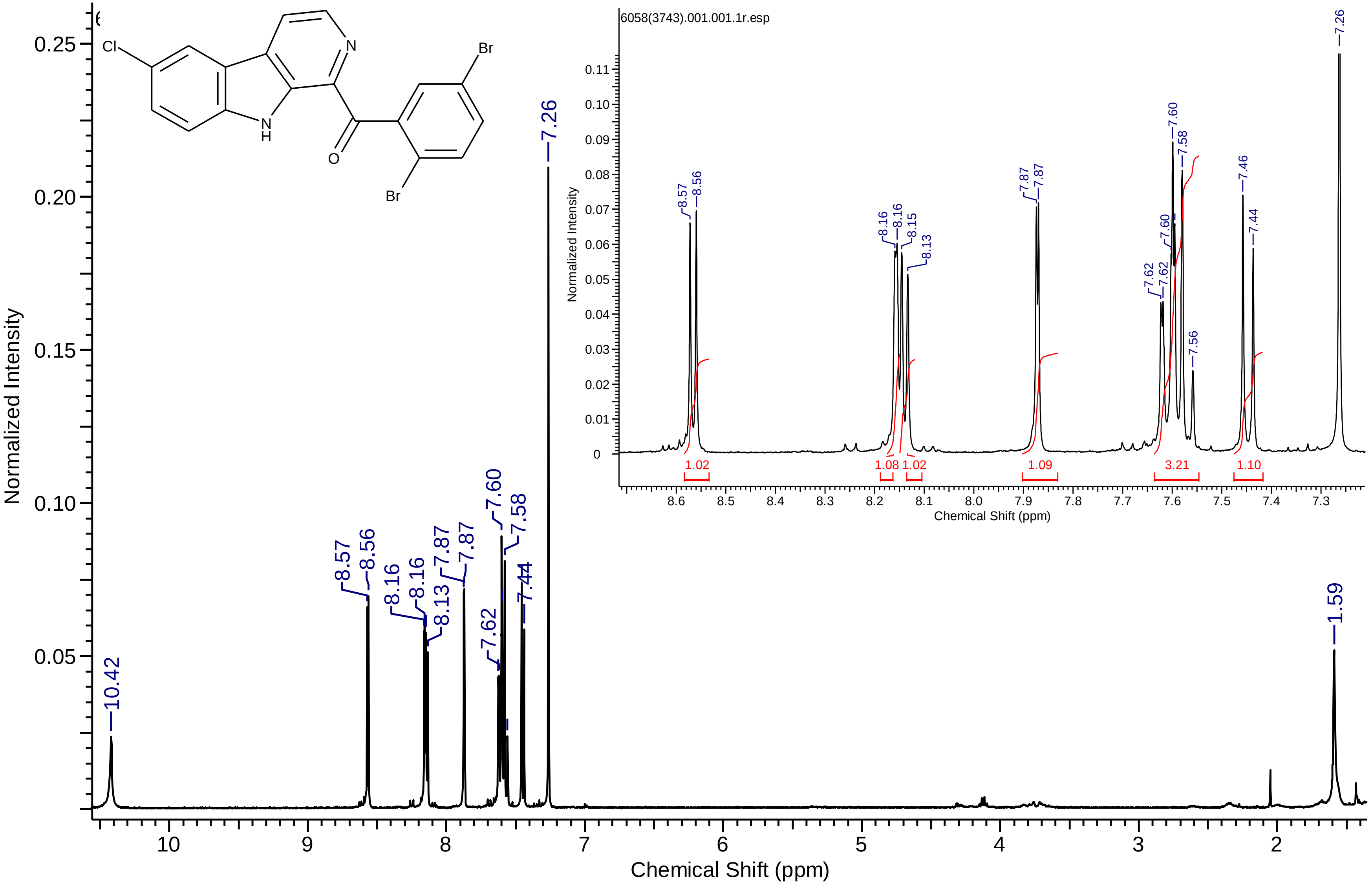

^{13}C NMR spectra of 6-chloro-1-(2’,5’-dibromobenzoyl)-β-carboline (20c).

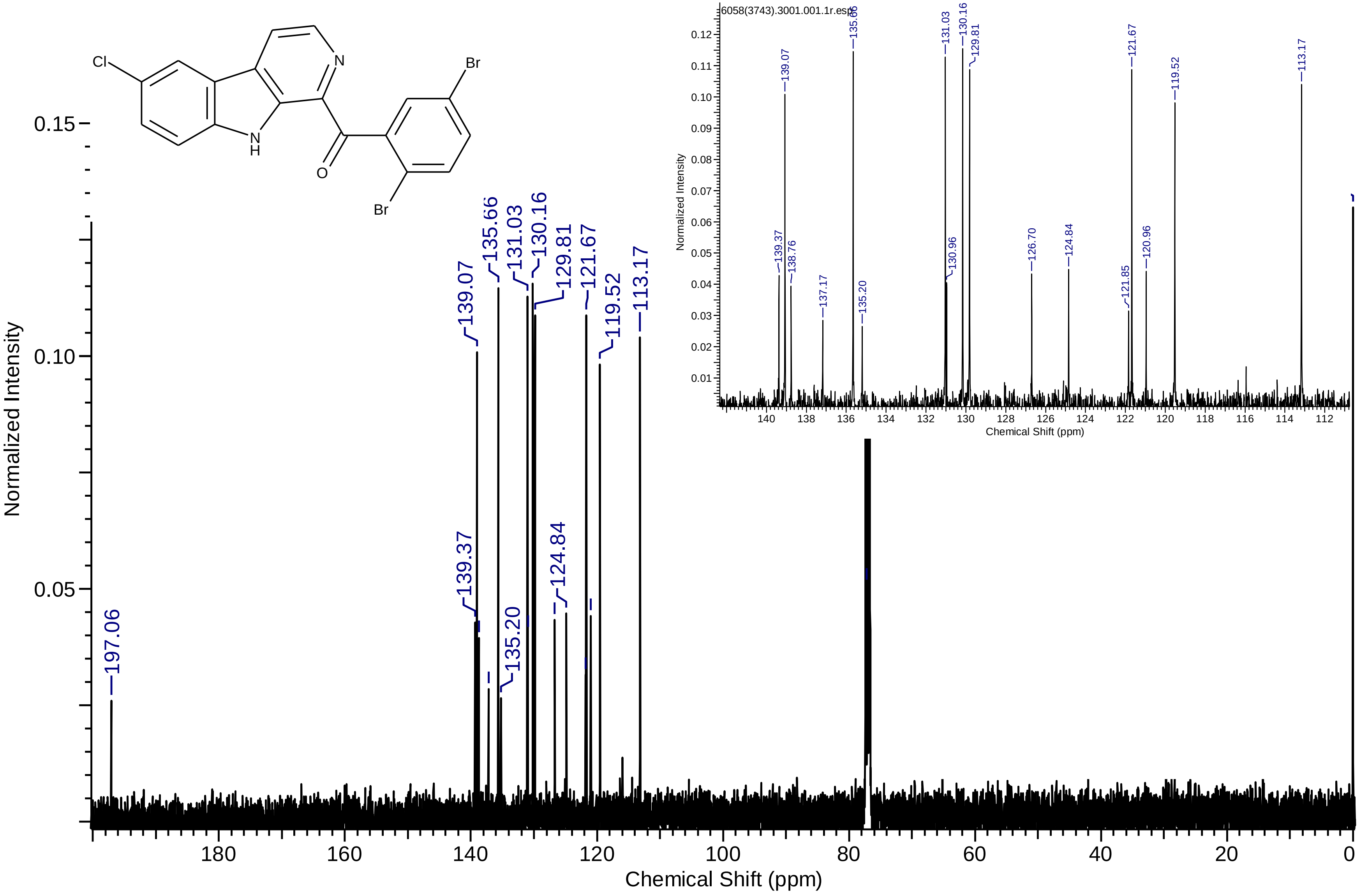

^{1}H NMR spectra of 6,8-dichloro-1-(2’,5’-dibromobenzoyl)- β-carboline (20d).

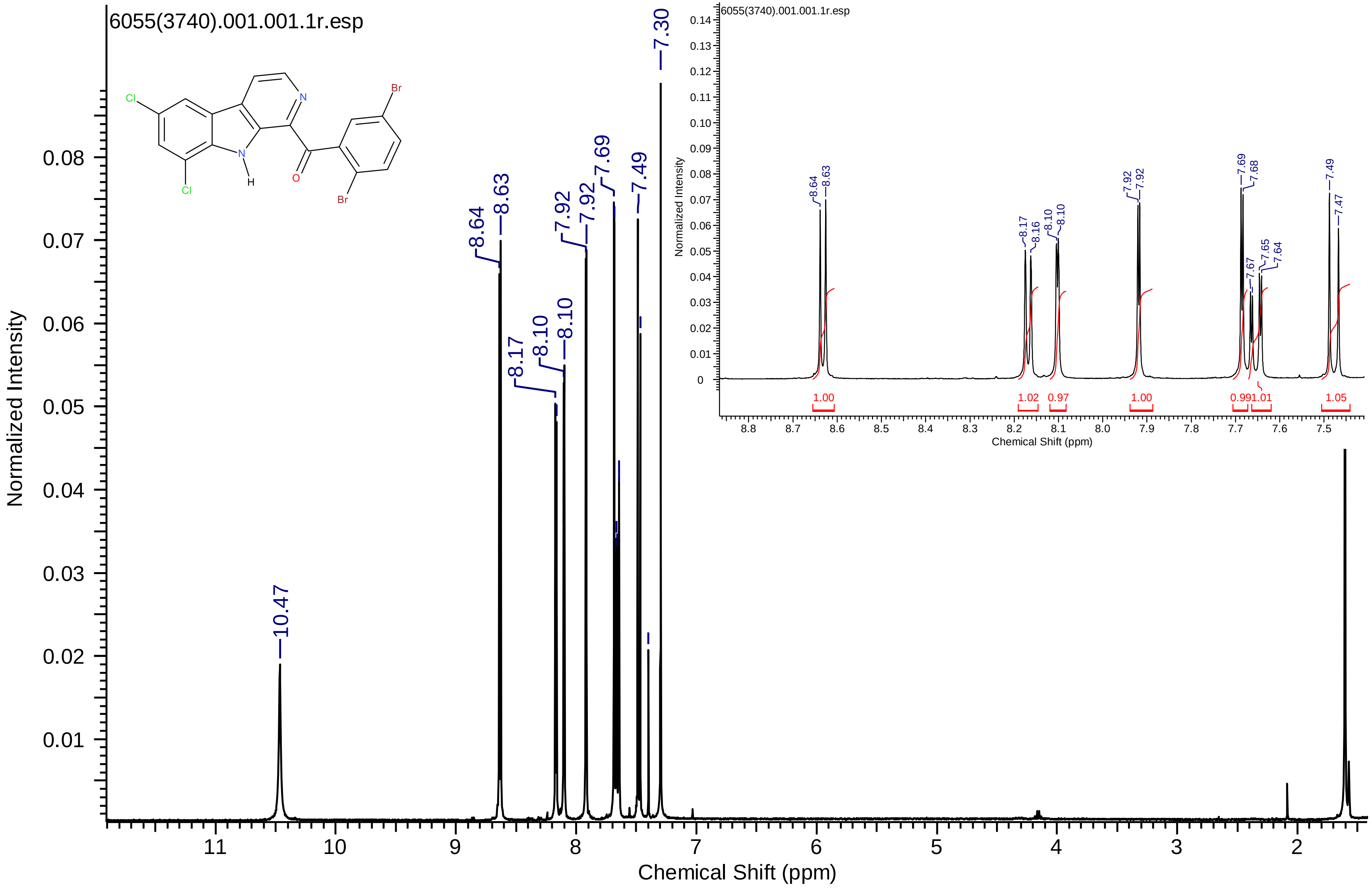

^{13}C NMR spectra of 6,8-dichloro-1-(2',5'-dibromobenzoyl)-β-carboline (20d).

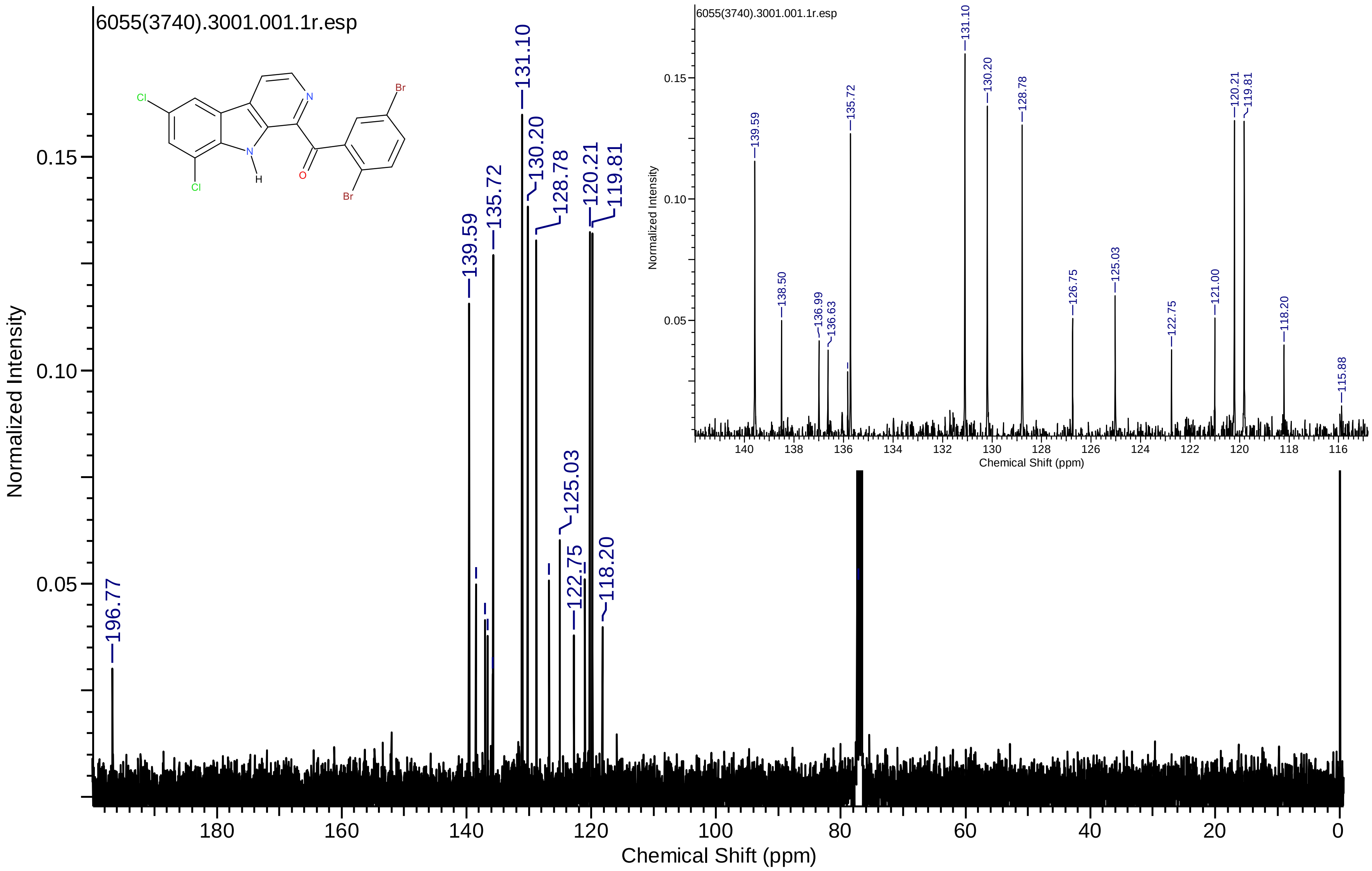

^{1}H NMR spectra of 3-bromofascaplysin (3).

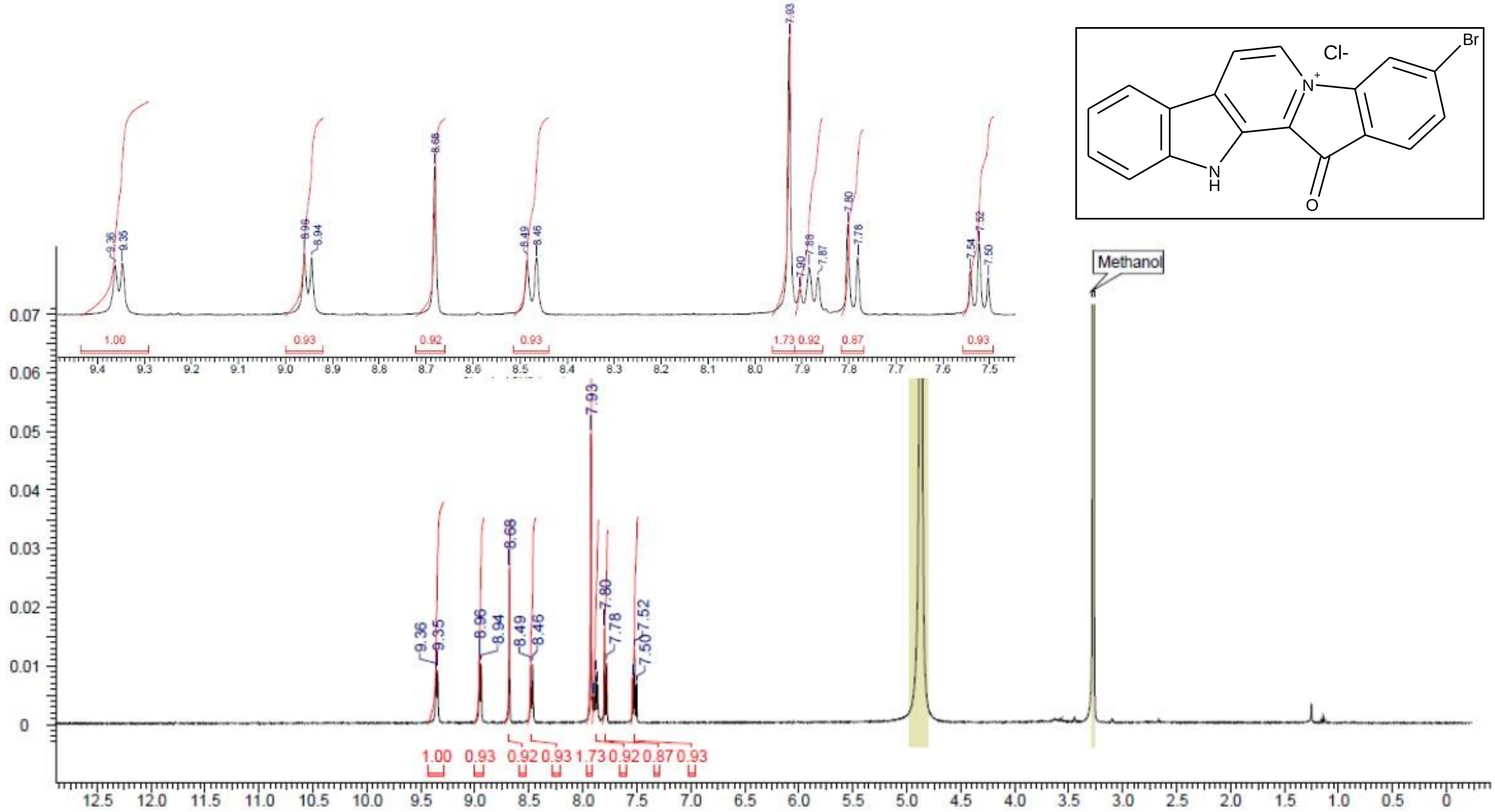

^{13}C NMR spectra of 3-bromofascaplysin (3).

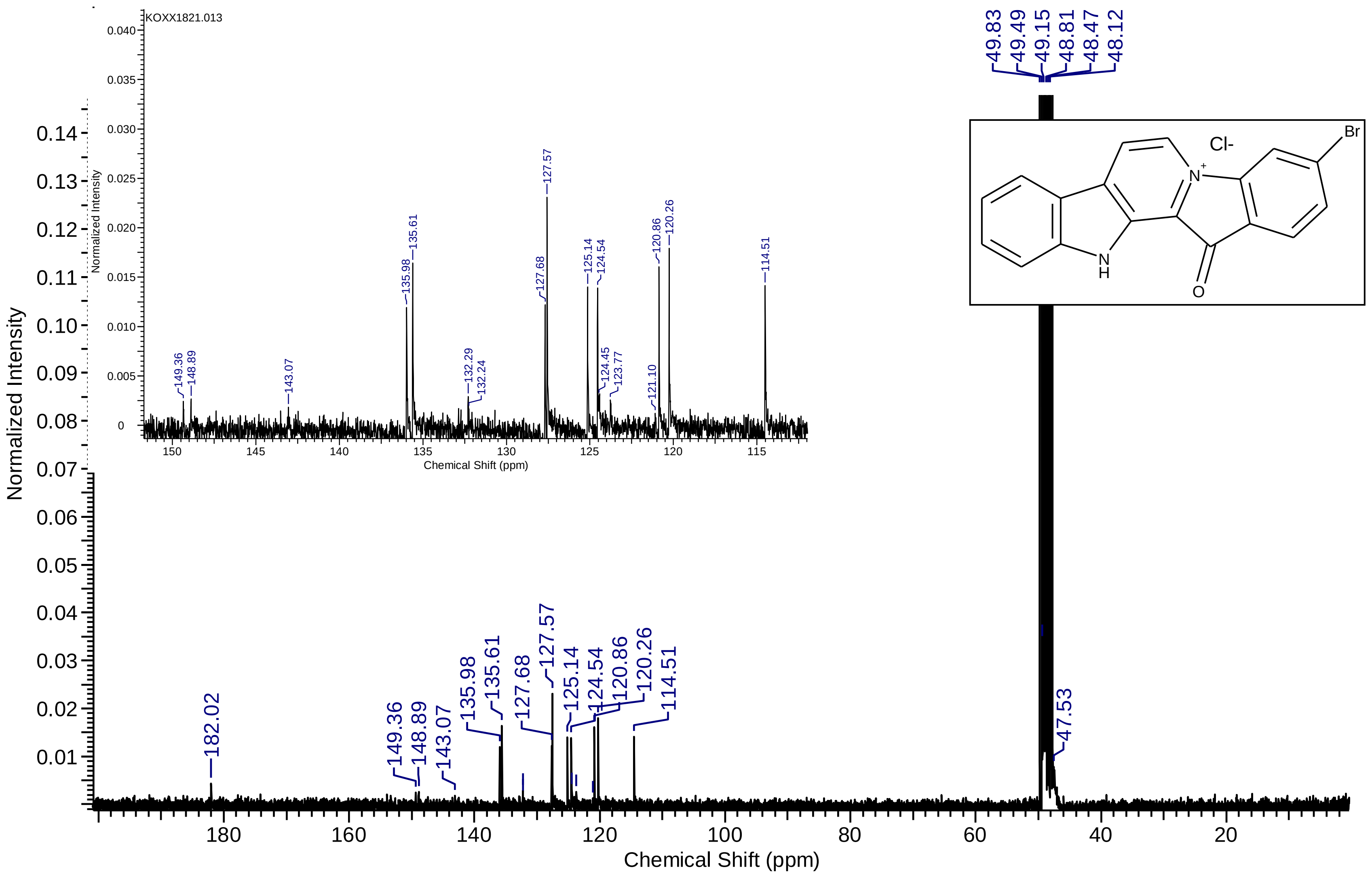

¹H NMR spectra of 12,13-dihydro-2,10-dibromo-13-oxopyrido[1,2-*a*:3,4-*b'*]diindol-5-ium chloride (13).

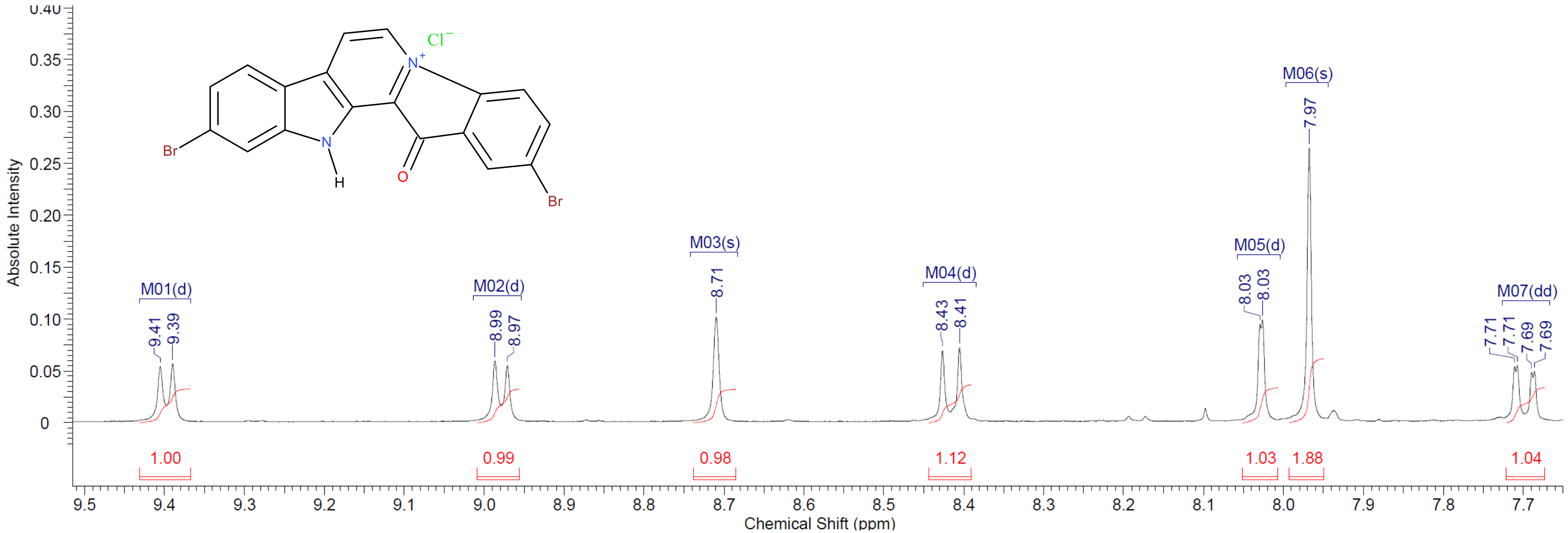

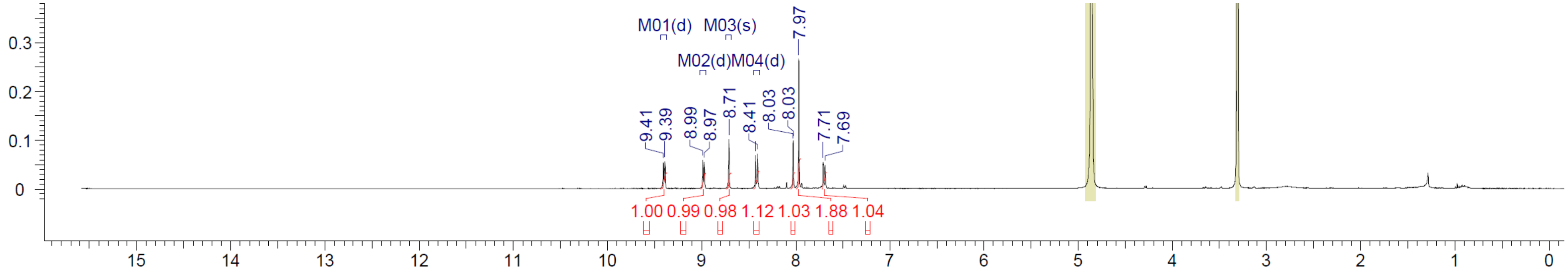

^{13}C NMR spectra of 12,13-dihydro-2,10-dibromo-13-oxopyrido[1,2-*a*:3,4-*b'*]diindol-5-ium chloride (13).

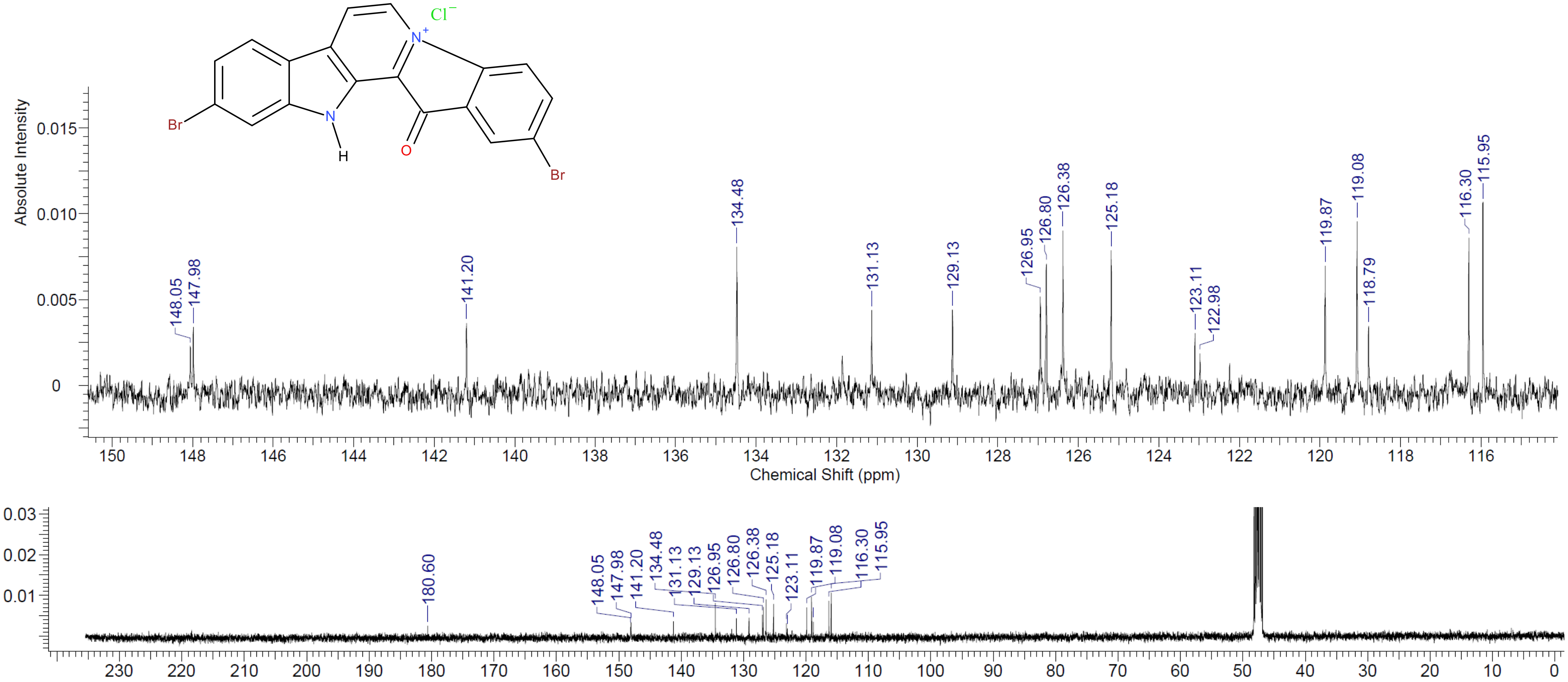

^{1}H NMR spectra of 3,10-dibromofascaplysin (5).

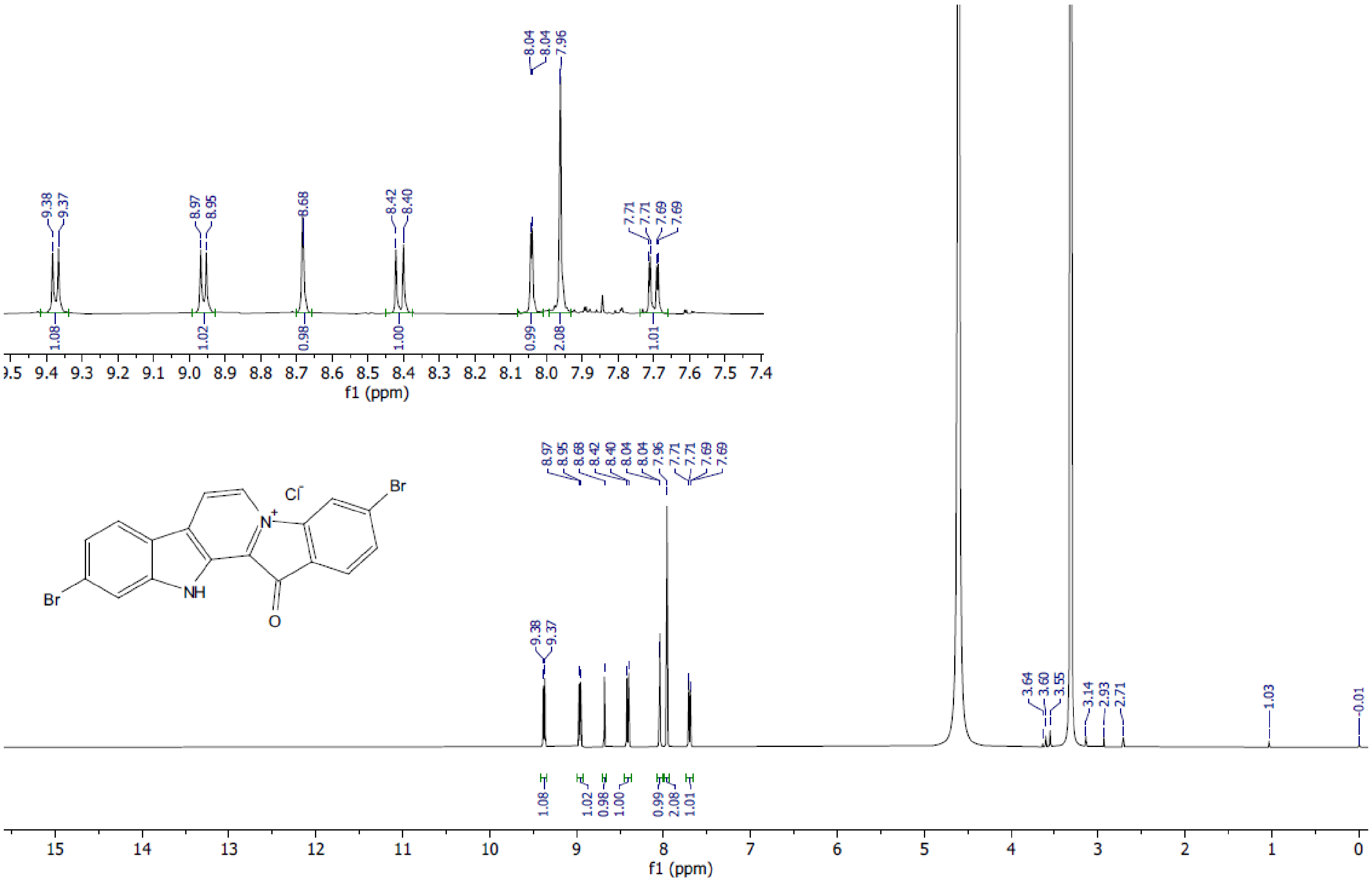

^{13}C NMR spectra of 3,10-dibromofascaplysin (5) in CD_3OD.

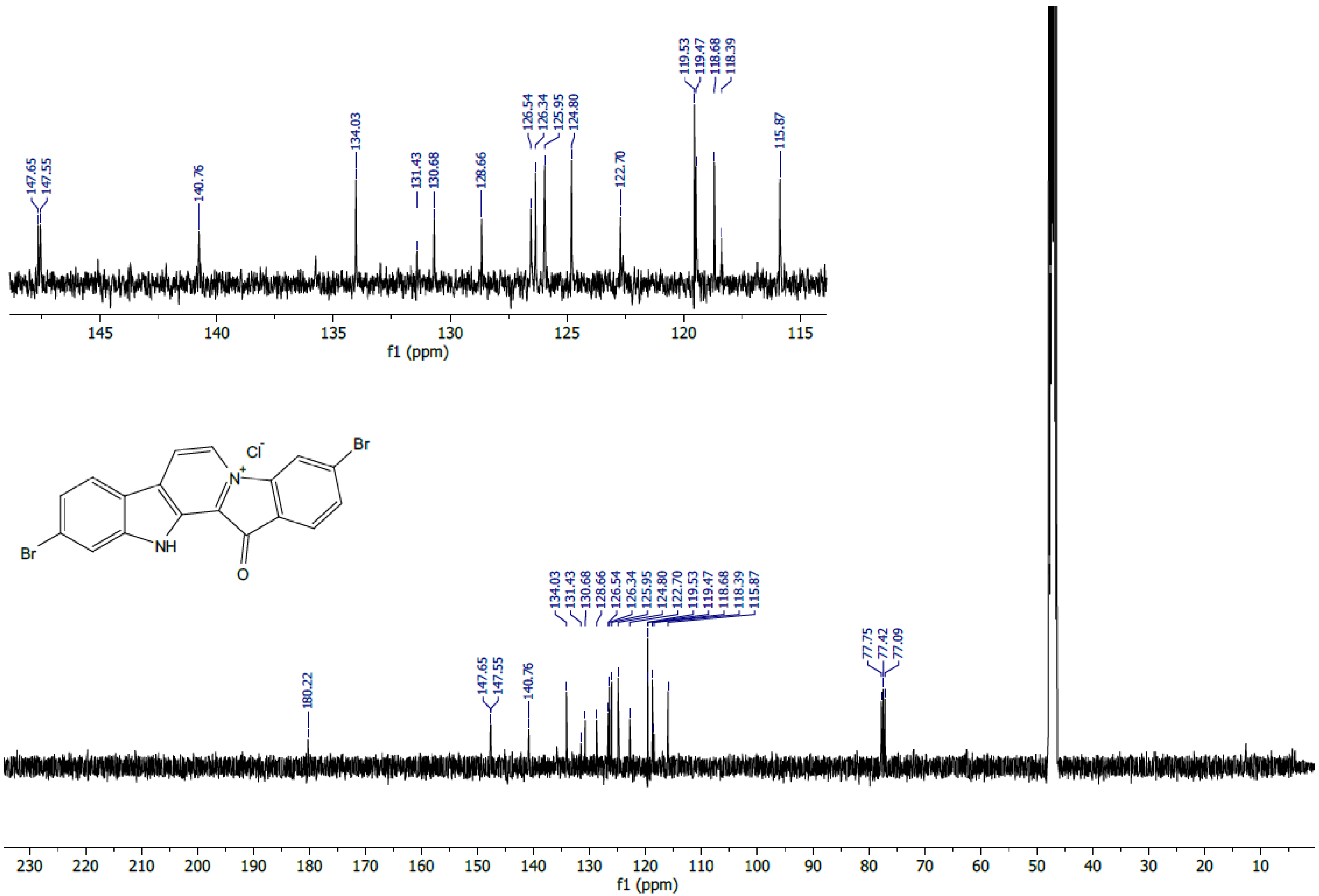

^{1}H NMR spectra of 12,13-dihydro-3.8-dibromo-13-oxopyrido[1,2-*a*:3,4-*b'*]diindol-5-ium chloride (14).

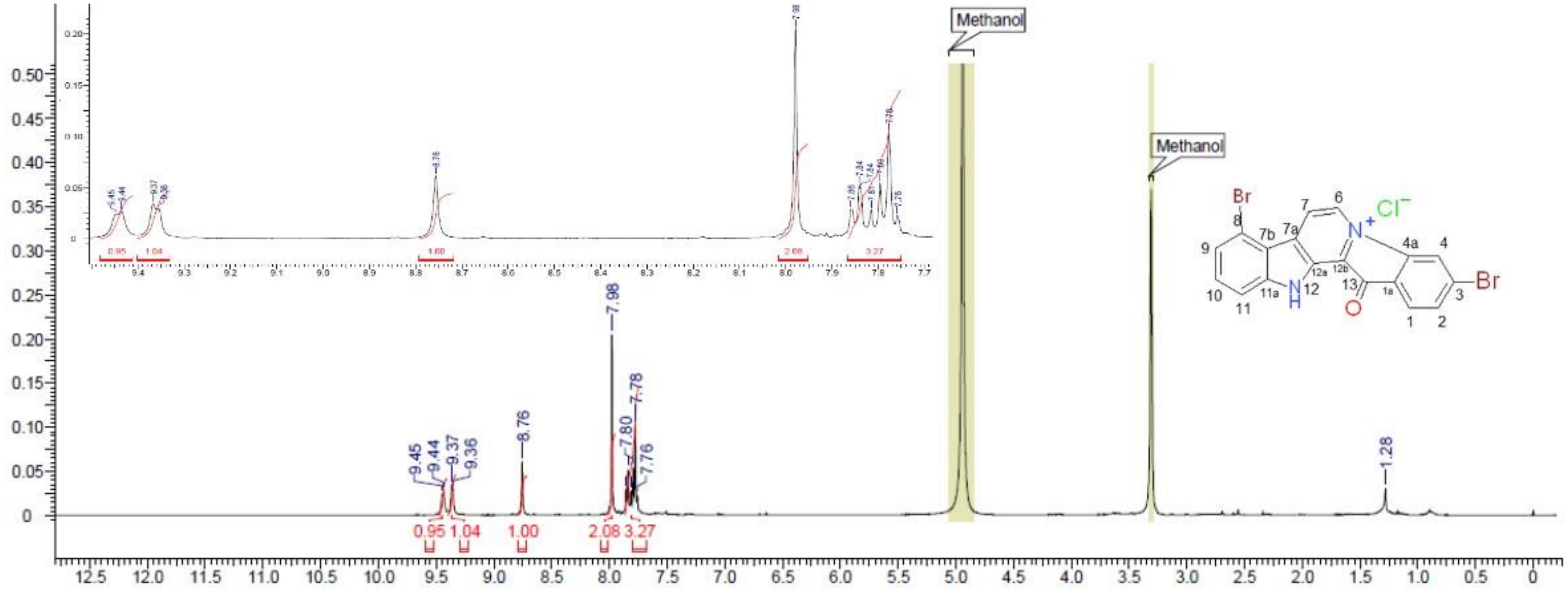

^{13}C NMR spectra of 12,13-dihydro-3.8-dibromo-13-oxopyrido[1,2-*a*:3,4-*b'*]diindol-5-ium chloride (14).

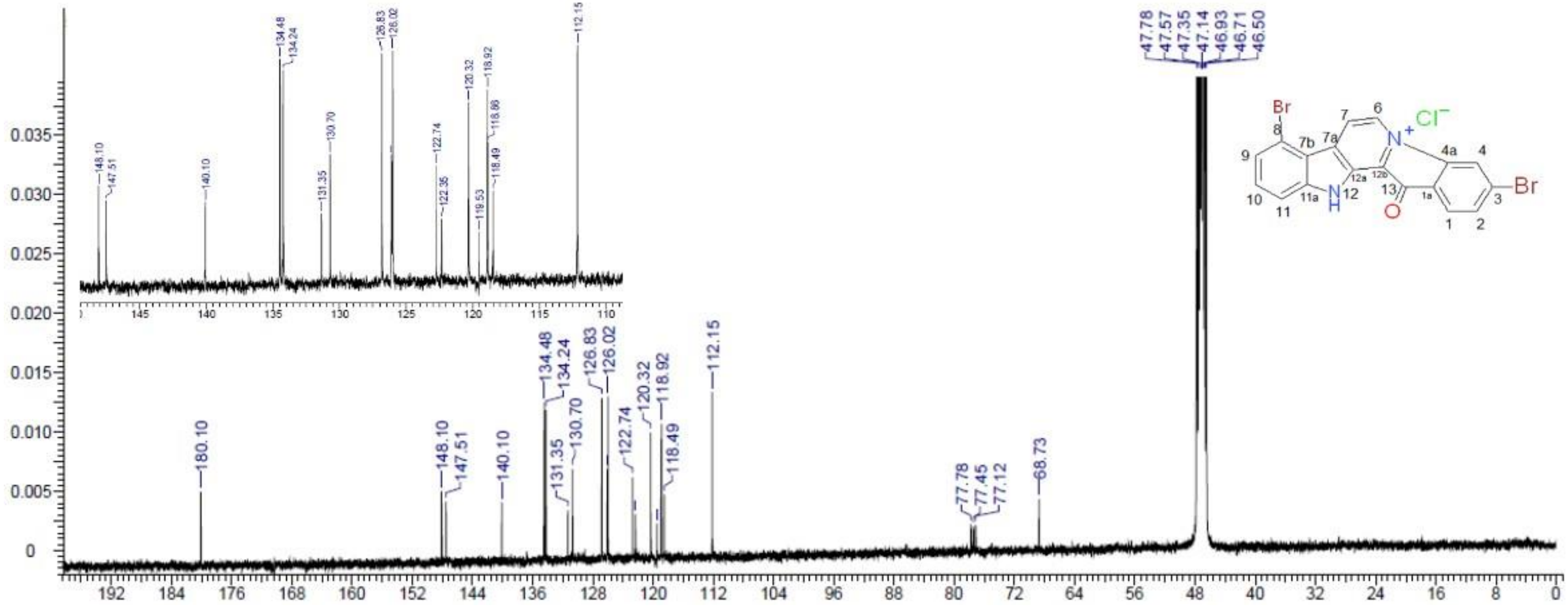

^{1}H NMR spectra of 12,13-dihydro-3.9-dibromo-13-oxopyrido[1,2-*a*:3,4-*b'*]diindol-5-ium chloride (21).

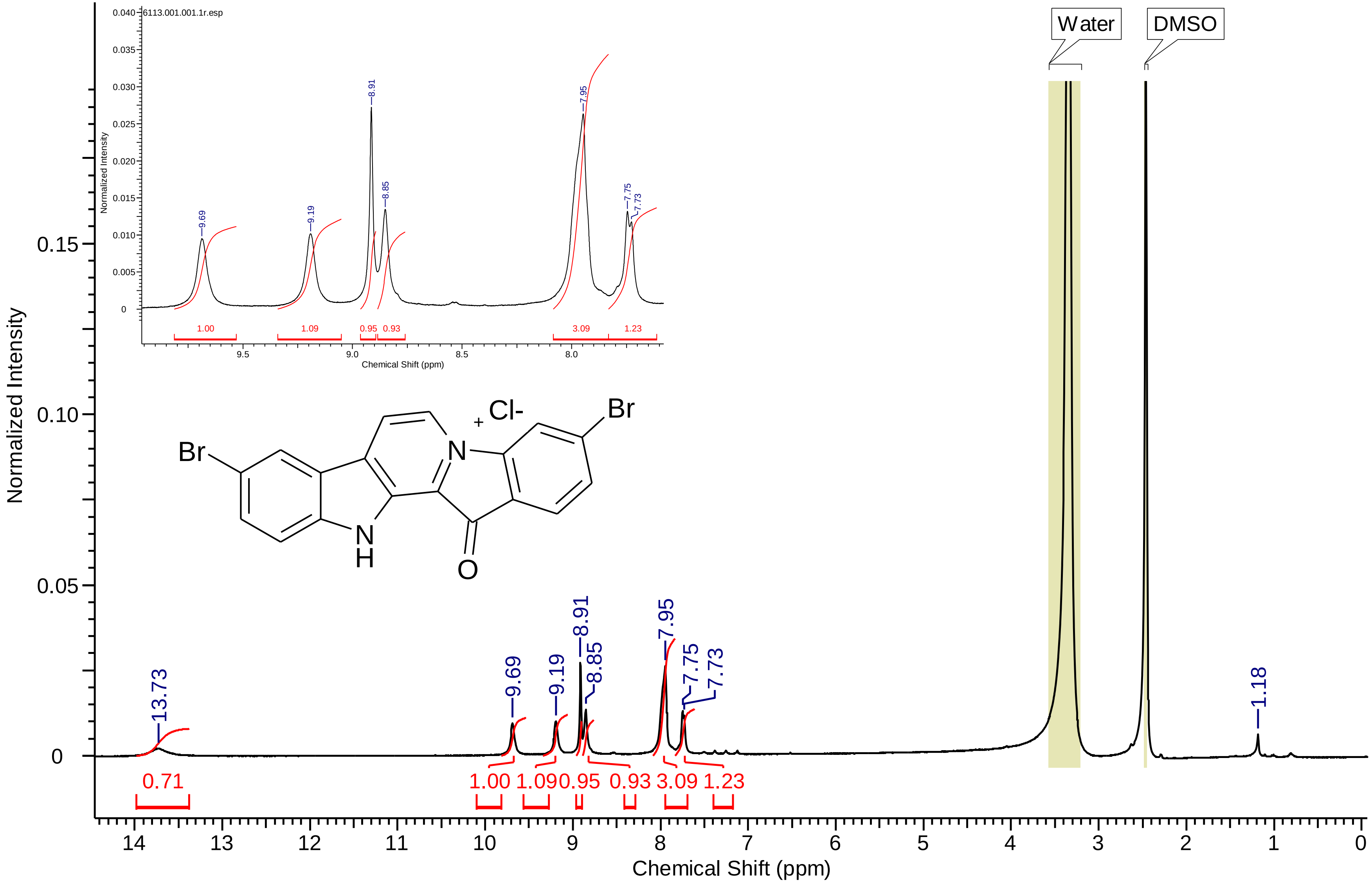

^{13}C NMR spectra of 12,13-dihydro-3.9-dibromo-13-oxopyrido[1,2-*a*:3,4-*b'*]diindol-5-ium chloride (21).

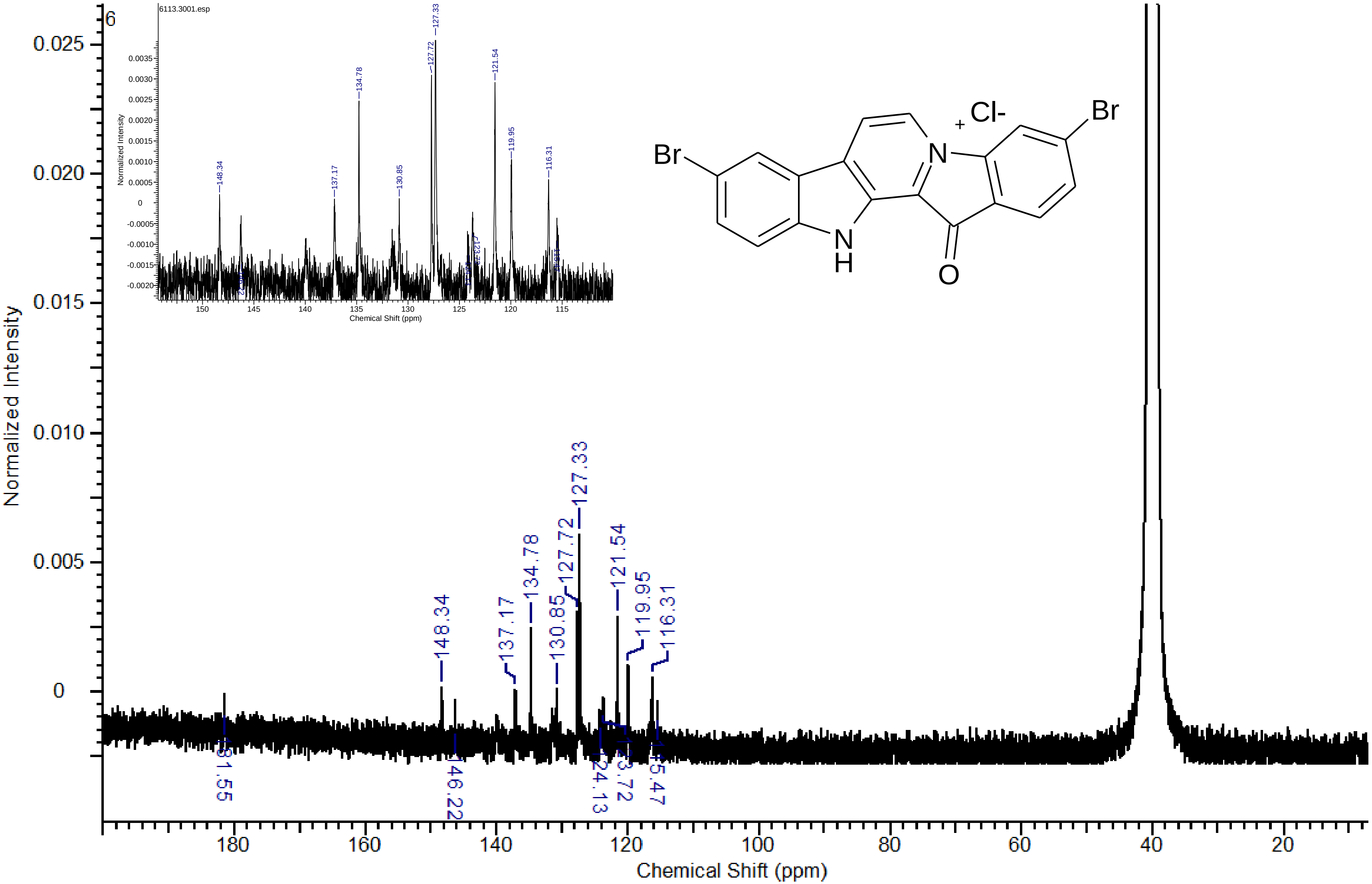

^{1}H NMR spectra of 12,13-dihydro-2.9-dibromo-13-oxopyrido[1,2-*a*:3,4-*b'*]diindol-5-ium chloride (22).

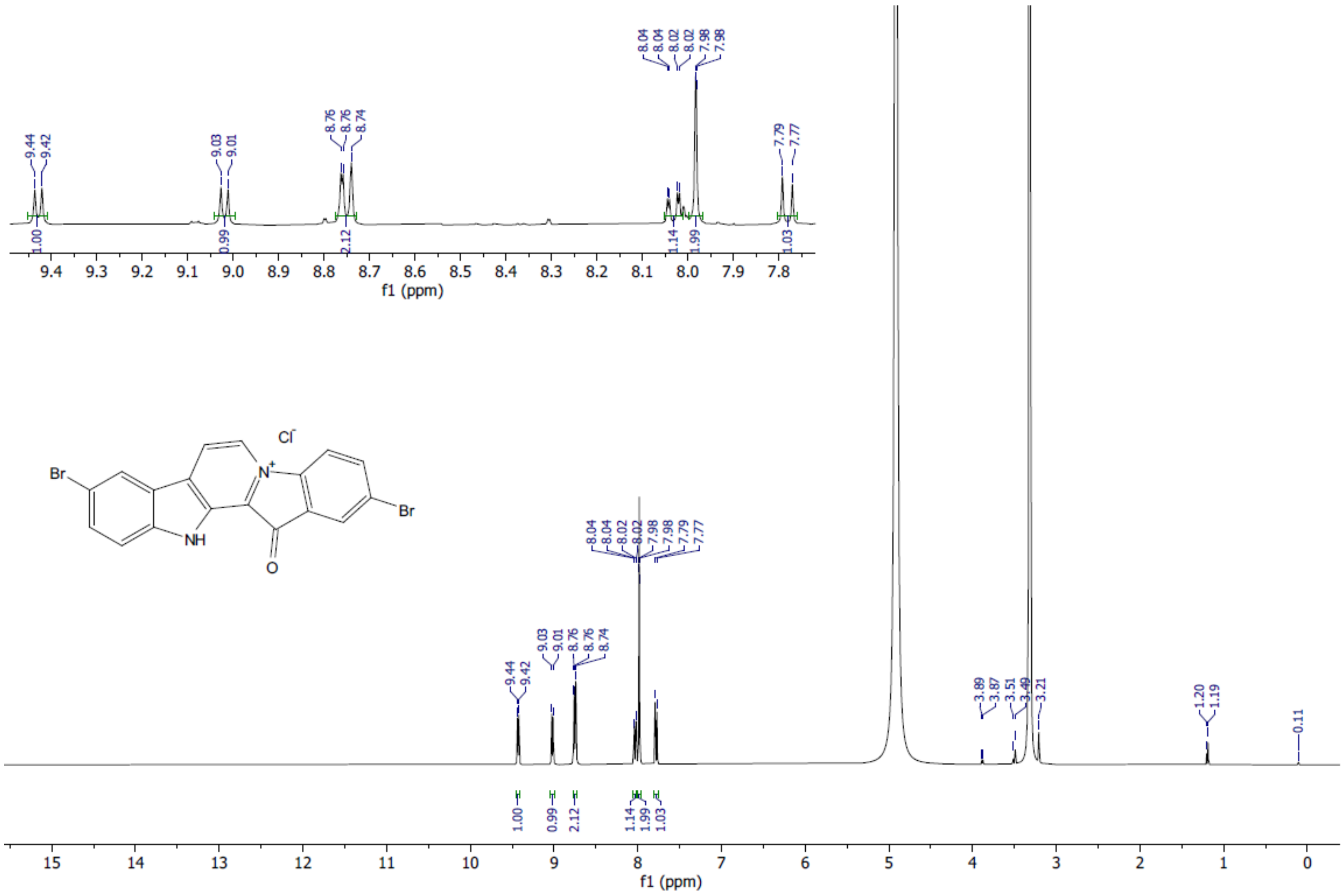

^{13}C NMR spectra of 12,13-dihydro-2.9-dibromo-13-oxopyrido[1,2-*a*:3,4-*b'*]diindol-5-ium chloride (22).

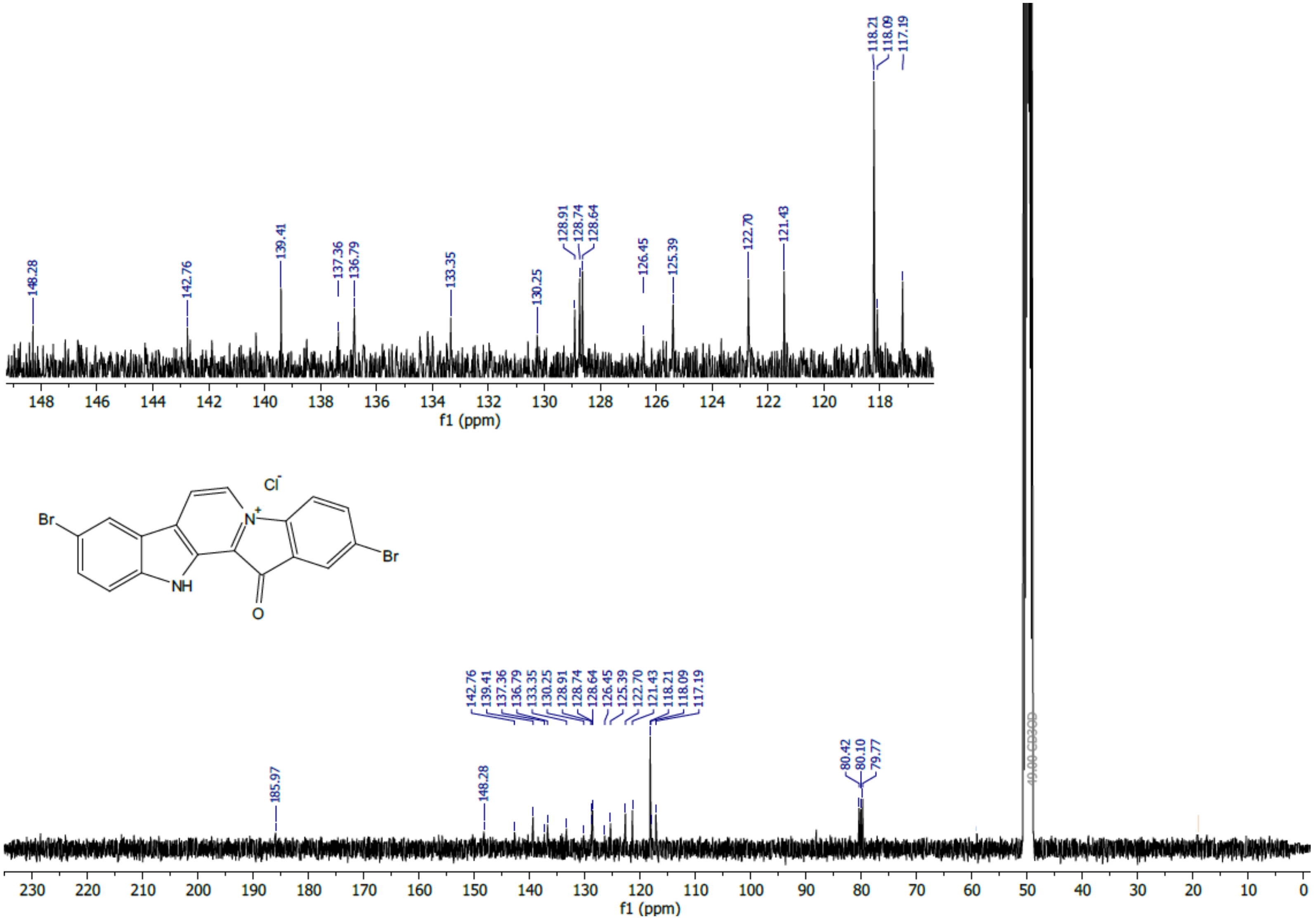

^{1}H NMR spectra of 12,13-dihydro-2-bromo-9-chloro-13-oxopyrido[1,2-*a*:3,4-*b*’]diindol-5-ium chloride (23).

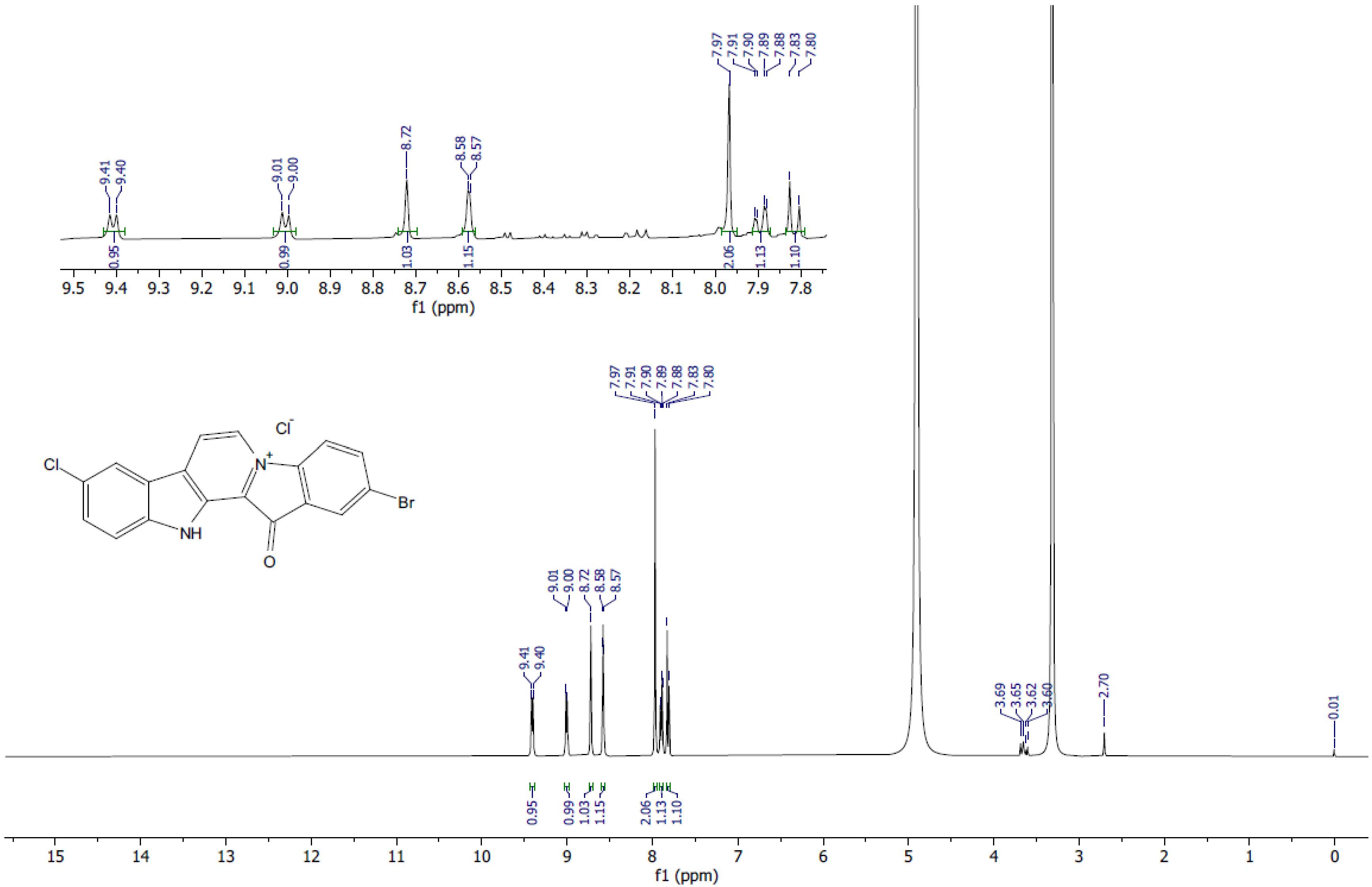

^{13}C NMR spectra of 12,13-dihydro-2-bromo-9-chloro-13-oxopyrido[1,2-*a*:3,4-*b'*]diindol-5-ium chloride (23).

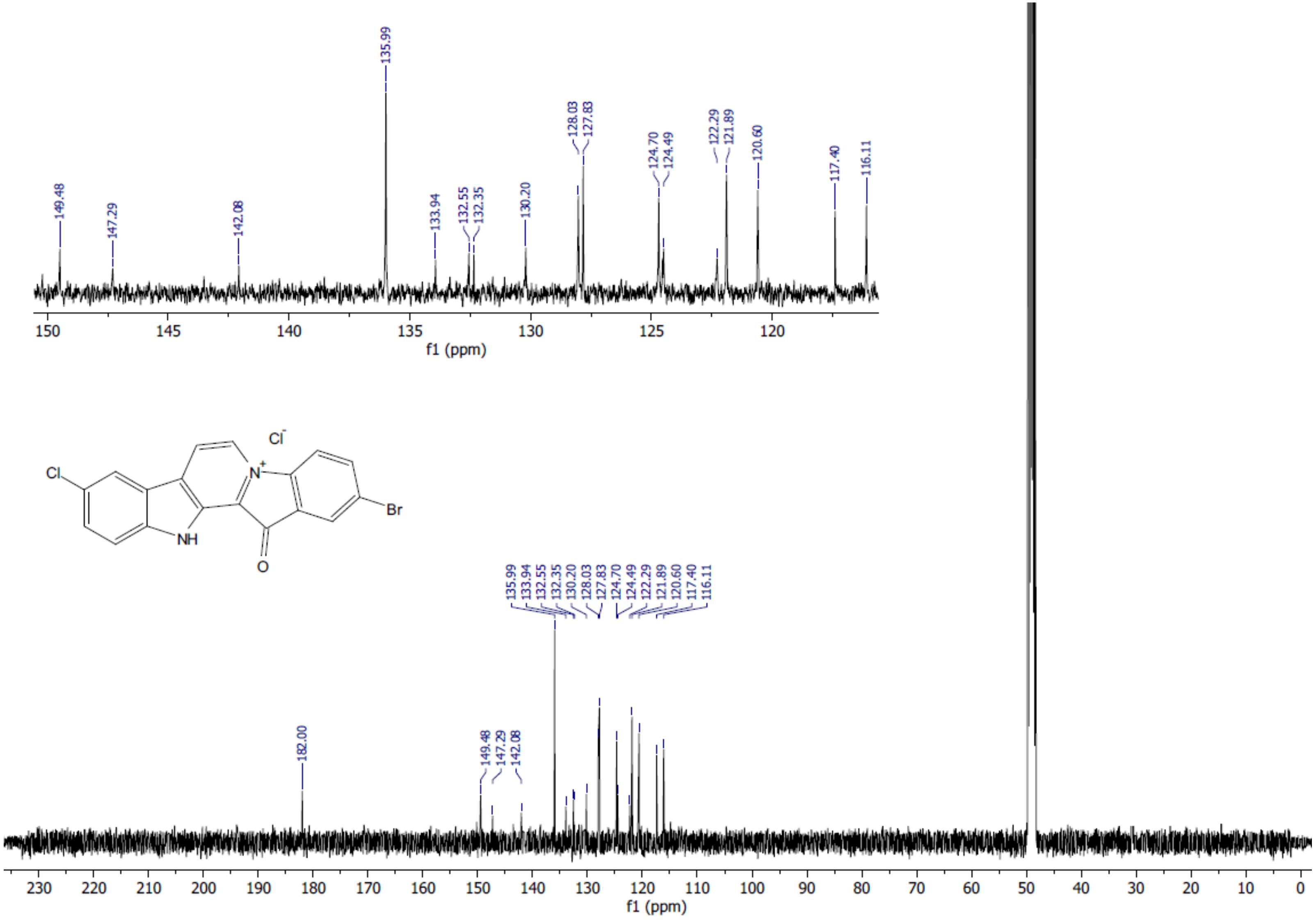

^{1}H NMR spectra of 12,13-dihydro-2-bromo-9.11-dichloro-13-oxopyrido[1,2-*a*:3,4-*b'*]diindol-5-ium chloride (24).

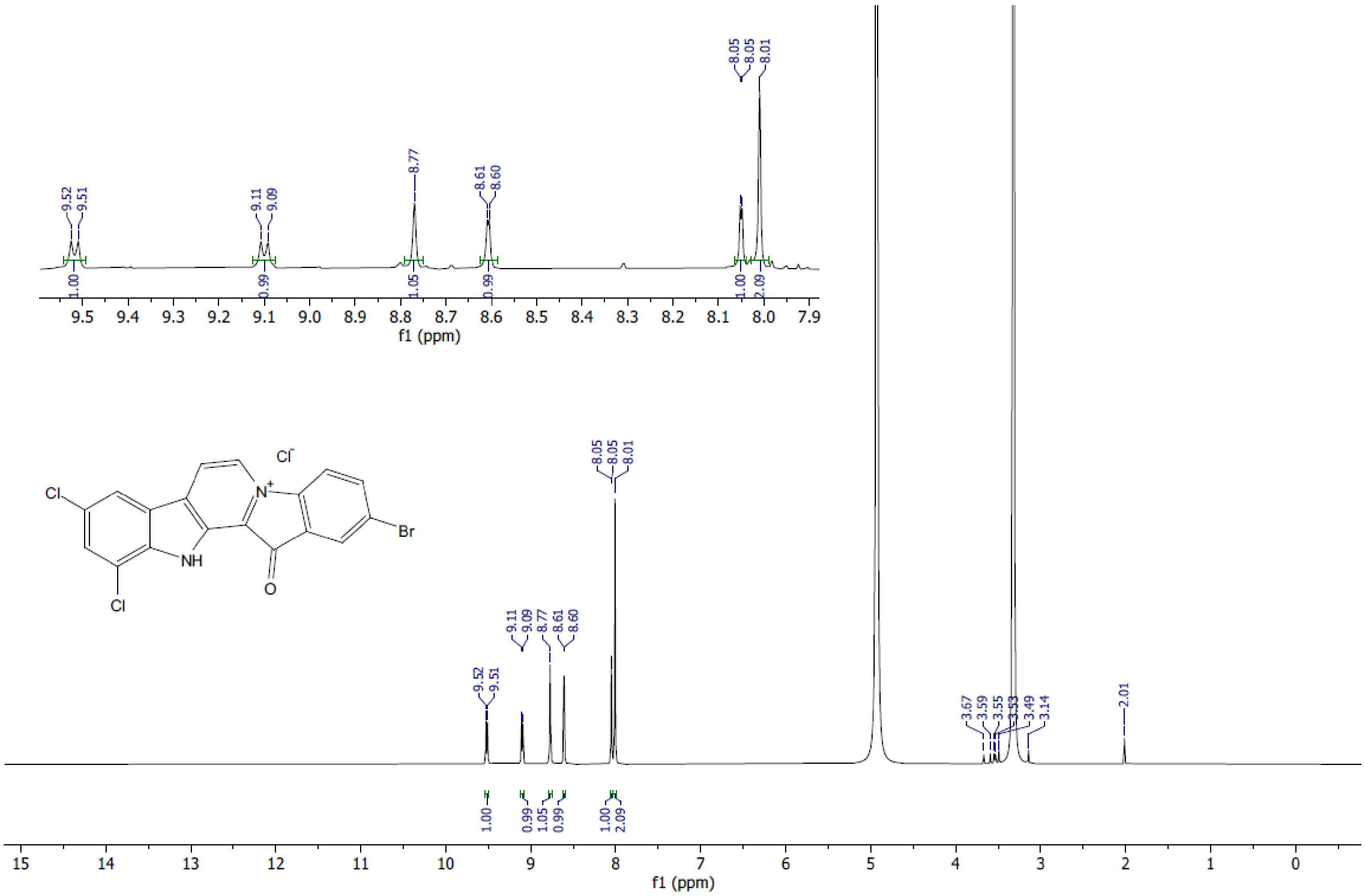

^{13}C NMR spectra of 12,13-dihydro-2-bromo-9.11-dichloro-13-oxopyrido[1,2-*a*:3,4-*b'*]diindol-5-ium chloride (24).

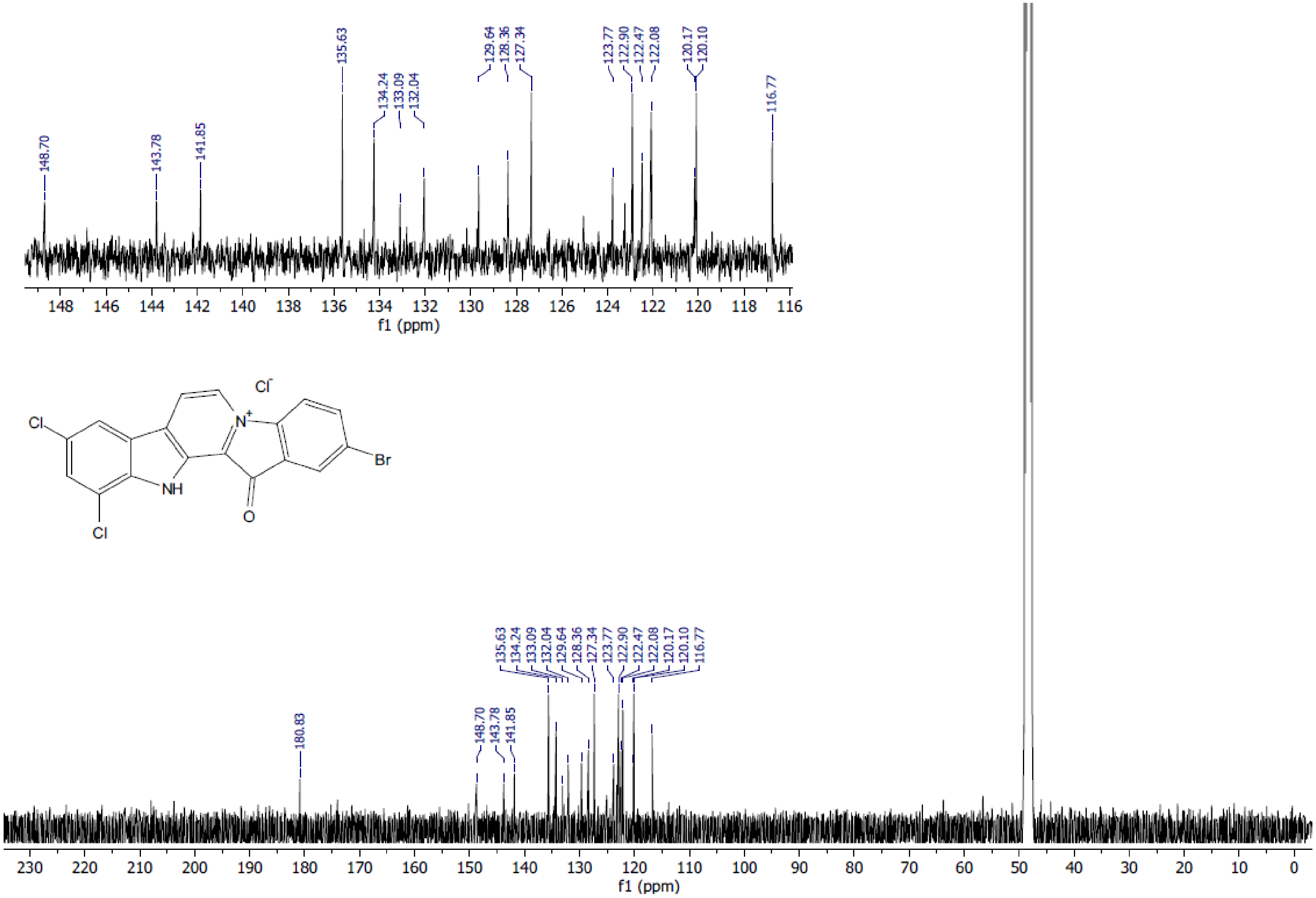

Supplement: Supplementary file 1 [file marinedrugs-23-00068-s001.zip › marinedrugs-3428146-supplementary.pdf]
